# Activation of AMPKα2 Is Not Crucial for Mitochondrial Uncoupling-Induced Metabolic Effects but Required to Maintain Skeletal Muscle Integrity

**DOI:** 10.1371/journal.pone.0094689

**Published:** 2014-04-14

**Authors:** Mario Ost, Franziska Werner, Janine Dokas, Susanne Klaus, Anja Voigt

**Affiliations:** German Institute of Human Nutrition (DIfE) Potsdam-Rehbruecke, Nuthetal, Germany; GDC, Germany

## Abstract

Transgenic (UCP1-TG) mice with ectopic expression of UCP1 in skeletal muscle (SM) show a phenotype of increased energy expenditure, improved glucose tolerance and increase substrate metabolism in SM. To investigate the potential role of skeletal muscle AMPKα2 activation in the metabolic phenotype of UCP1-TG mice we generated double transgenic (DTG) mice, by crossing of UCP1-TG mice with DN-AMPKα2 mice overexpressing a dominant negative α2 subunit of AMPK in SM which resulted in an impaired AMPKα2 activity by 90±9% in SM of DTG mice. Biometric analysis of young male mice showed decreased body weight, lean and fat mass for both UCP1-TG and DTG compared to WT and DN-AMPKα2 mice. Energy intake and weight-specific total energy expenditure were increased, both in UCP1-TG and DTG mice. Moreover, glucose tolerance, insulin sensitivity and fatty acid oxidation were not altered in DTG compared to UCP1-TG. Also uncoupling induced induction and secretion of fibroblast growth factor 21 (FGF21) from SM was preserved in DTG mice. However, voluntary physical cage activity as well as *ad libitum* running wheel access during night uncovered a severe activity intolerance of DTG mice. Histological analysis showed a progressive degenerative morphology in SM of DTG mice which was not observed in SM of UCP1-TG mice. Moreover, ATP-depletion related cellular stress response via heat shock protein 70 was highly induced, whereas capillarization regulator VEGF was suppressed in DTG muscle. In addition, AMPKα2-mediated induction of mitophagy regulator ULK1 was suppressed in DTG mice, as well as mitochondrial respiratory capacity and content. In conclusion, we demonstrate that AMPKα2 is dispensable for SM mitochondrial uncoupling induced metabolic effects on whole body energy balance, glucose homeostasis and insulin sensitivity. But strikingly, activation of AMPKα2 seems crucial for maintaining SM function, integrity and the ability to compensate chronic metabolic stress induced by SM mitochondrial uncoupling.

## Introduction

Skeletal muscle (SM) as a major body compartment is responsible for about 20% of resting energy expenditure and up to 90% of the energy expenditure during physical activity. It is thus an important determinant of overall substrate metabolism. The metabolic syndrome is closely associated with altered glucose and lipid metabolism in SM [1]. Conversely, muscle specific ectopic expression of uncoupling protein 1 (UCP1), the mitochondrial uncoupling protein of brown adipose tissue (BAT), leads to increased energy expenditure, delayed diet-induced obesity development, improved glucose homeostasis, and increased longevity in these UCP1-TG mice [2]–[4]. UCP1-TG mice show an increased substrate flux through the glycolytic pathway paralleled by increased insulin-stimulated glucose uptake and increased lipid metabolism in skeletal muscle [3]–[5]. Most importantly, in two different transgenic mouse models it was shown that UCP1 expression in skeletal muscle led to an increased phosphorylation of AMP-activated protein kinase (AMPK) [4], [6], thus linking the positive metabolic effects of skeletal muscle uncoupling to activation of AMPK.

As an intracellular energy sensor, AMPK is considered the master regulator of cellular energy homeostasis [7]. AMPK is a heterotrimeric serine/threonine kinase which is activated by metabolic stress, often in response to an increased AMP/ATP ratio [8], [9]. Essential for AMPK activation is the phosphorylation of Thr172 of the catalytic α-subunit [10]. In skeletal muscle, AMPK is an important regulator of glucose uptake during exercise [11]. Muscle AMPK is involved in the coordinated transcription of genes important for lipid and glucose metabolism during exercise and for acute control of metabolic fluxes, namely the switch from carbohydrate to lipid oxidation [9], [12]–[15]. In response to endurance exercise training, AMPK appears to be important for glycolytic to oxidative fiber-type switch [16], [17]. AMPK regulates basal VEGF expression and capillarization in muscle [18]. In addition, mice lacking the AMPK activating upstream kinase LKB1 or both AMPKβ subunits showed dramatically impaired exercise tolerance as well as reduced mitochondrial content and capacity [19], [20].

Additionally, AMPK is viewed as an important regulator of integrated signaling networks and stress resistance [21], [22] which also contributes to long term regulation of muscle turnover by decreasing protein synthesis and activating autophagy [23]. There is growing evidence that autophagy is a central cellular control mechanism removing damaged proteins and organelles such as mitochondria to maintain myofiber integrity and energy metabolic homeostasis (reviewed in Ref. [24]). Impaired function of SM autophagy was not only shown to be related to muscle dystrophy and stress response activation [25], [26] but also coupled to AMPK function [27]. Recently, we could show that autophagy machinery is induced in mitochondrial uncoupled SM of UCP1-TG [28], suggesting an important role of AMPK as molecular checkpoint regulating both energy homeostasis and cellular function.

Here we aimed to explore the role of AMPKα2 activation in the beneficial metabolic effects of skeletal muscle uncoupling. To this effect UCP1-TG mice were crossbred with transgenic mice expressing a mutated, dominant negative “kinase-dead” form of the AMPKα2 subunit which is the dominant α-subunit in SM [11]. In these DN-AMPK mice AMPKα2 can still be phosphorylated but basal and stimulated AMPK activities are several fold decreased [29], [30]. Using double transgenic (DTG) mice with both SM UCP1 expression and AMPKα2 ablation we thus addressed two questions: (I) What is the role of AMPKα2 in mitochondrial uncoupled SM on regulation of whole body energy balance, glucose homeostasis and insulin sensitivity? (II) How is muscle function and performance of UCP1-TG mice affected by inactivation of AMPKα2?

## Methods

### Animals and experimental setup

Transgenic DN-AMPKα2 mice on a C57BL/6J background [11] were kindly provided by Prof. Morris Birnbaum from the University of Pennsylvania in Philadelphia (USA). They were crossed with heterozygous HSA-UCP1 transgenic mice on a C57BL/6J background with ectopic expression of UCP1 in skeletal muscle [31] to obtain 4 genotypes: wildtype (WT), DN-AMPKα2, UCP1-TG, and UCP1-TG/DN-AMPKα2 (double transgenic, DTG). All experiments were performed with male mice (9–10 per genotype). Mice were weaned at 3 weeks of age, single caged, and fed standard chow with food and water supplied ad libitum. At 10 weeks of age, an oral glucose tolerance test was performed. Mice were supplied a running wheel for measurement of voluntary activity in the 11^th^ week of age. Simultaneously, physical activity was monitored using infrared motion detectors (both from TSE Systems GmbH, Germany) and food intake was recorded. At 12 weeks of age, mice were euthanized (isoflurane) in the morning, 3 hrs after food withdrawal, plasma and tissue samples were collected, immediately frozen in liquid nitrogen, and stored at −80°C until further analysis. An additional subset of older aged mice was used for functional analysis of *ex vivo* skeletal muscle glucose transport and fatty acid oxidation at 12–17 weeks of age and *ex vivo* skeletal muscle mitochondrial function at 17–23 weeks of age, to be able to compare the data with prior functional studies from our group [5], [32].

### Ethics Statement

Animal maintenance and experiments were approved by the animal welfare committee of the Ministry of Agriculture and Environment (State of Brandenburg, Germany, No. 23-2347-16-2010).

### Phenotyping and physical activity

Body weight and composition was determined weekly using quantitative magnetic resonance (QMR) (Bruker's Minispec MQ10, Housten Texas, USA) as described [3]. Energy expenditure of single mice was measured by indirect calorimetry over 24 h using an open respirometric system at 11 week of age as described before [2]. Mice were kept in normal housing cages and had free access to food and water during the measurement. Locomotor activity was assessed by parallel measurement of spontaneous physical activity using an infrared method and running wheel activity providing mice continuous voluntary access to a running wheel counting wheel rotations (both TSE Systems GmbH, Germany) in the same cages.

### Oral glucose tolerance test (oGTT) and plasma analyses

An oGTT was performed at 10 weeks of age. Glucose (2 mg/g body weight, 20% solution) was applied orally three hours after food withdrawal. Insulin levels were measured before, 15, and 30 min after oral glucose application. Blood glucose was determined in tail blood using a common glucose sensor (Bayer, Germany), and plasma insulin was measured by an ultra-sensitive ELISA assay (ALPCO Diagnostics, USA). Plasma FGF21 was measured using a mouse/rat FGF-21 Quantikine ELISA Kit (R&D Systems).

### Measurement of AMPK activity

AMPK activity was measured by incorporation of ^32^P into a synthetic SAMS peptide, a specific AMPK substrate [33]. *Gastrocnemius* muscle was first homogenized in ice-cold lysis buffer containing 50 mM HEPES, 10% glycerol (v/v), 1 mM EDTA and 1% Triton. Catalytic subunits of AMPK were immunoprecipitated by Dynabeads Protein G (Invitrogen, Germany) coupled anti-AMPKα1 (Millipore) and anti-AMPKα2 (Santa Cruz, USA) for 2 h at 4°C. The AMPK assay was performed as described [34]. Briefly, the reaction was initiated by the addition of γ^32^P-ATP/Mg to assay buffer containing 200 µM AMP, 200 µM SAMS peptide and 0.2% Triton. After 15 min incubation at 37°C, the reaction was stopped by spinning down of the beads, and 15 µl of the supernatant was spotted onto phosphocellulose paper (P81, Whatman), which was suspended in 1% orthophosphoric acid. P81 papers were washed 3 times in 1% acid and finally in water, blow-dried, and radioactivity was counted in a standard scintillation counter.

### Analysis of glucose transport and fatty acid oxidation

Ex vivo assays were performed essentially as described [5], [35]–[37]. Briefly, mice were fasted for 4 hrs prior to the study. To asses glucose uptake *Extensor digitorum longus* (EDL) muscles were removed from anesthetized mice (Avertin, 2,2,2-tribromo ethanol 99% and tertiary amyl alcohol, at 20 µl/g body weight i.p) and incubated for 30 min at 30°C in vials containing pre-gassed (95% O_2_/5% CO_2_) Krebs-Henseleit buffer (KHB, 5 mM HEPES), supplemented with 15 mM mannitol and 5 mM glucose. After recovery, EDL muscle were placed into new vials and incubated for 30 min in KHB/5 mM HEPES/15 mM mannitol/5 mM glucose under basal condition or in the presence of 120 nM insulin (Actrapid, Novo Nordisk, Mainz, Germany) throughout the duration of the glucose transport experiment. Thereafter, muscles were transferred to new vials containing pre-oxygenized KHB supplemented with Insulin and 15 mM mannitol and incubated for 10 min. Finally, muscles were transferred to new vials containing KHB supplemented with 1 mM [^3^H]2-desoxy-glucose (2.5 mCi/ml) and 19 mM [^14^C]mannitol (0.7 mCi/ml) for 20 min. Muscle were immediately frozen in liquid nitrogen and stored at -80°C for subsequent analysis. Cleared cytosolic lysate were used to determine incorporated radioactivity by scintillation counting. To evaluate palmitate oxidation, *Soleus* muscles were first pre-incubated in pre-gassed KHB containing 5 mM glucose, 15 mM mannitol and 3.5% fatty acid-free BSA for 15 min. Subsequently, muscles were transferred to new vials containing freshly pre-gassed KHB with 4 mCi/ml [3H]palmitate and 600 µM unlabeled palmitate at 30°C for 2 hrs with or without 2 mM AICAR (5-Aminoimidazole-4-carboxamide 1-β-D-ribo-furanoside, Biomol International). After absorption of fatty acids to activated charcoal fatty acid oxidation was determined by measuring tritiated water using a scintillation counter. All incubation steps were conducted under slight agitation and constant gassing (95% O_2_/5% CO_2_) at 30°C.

### Citrate Synthase activity

Citrate Synthase (CS) activity was determined spectrometrically as described previously [38] by monitoring the formation of DTNB at 412 nm. Briefly, *Quadriceps* muscle tissue was homogenized in 50 mM Tris, 1 mM EDTA (pH 7.4) and 0.1% Triton X-100 and centrifuged at 13000 g for 10 minutes at 4°C. The supernatant was used to determine the protein content and CS activity levels. 10 µl of 1∶6 diluted tissue extract was loaded into one well of a 96-well plate. Afterwards, 215 µl reaction buffer (100 mM Tris, 1 mM MgCl2, 1 mM EDTA (pH 8.2) 0.1 M DTNB), 25 µl Acetyl CoA (3.6 mM) were added. All analyses were completed in triplicates. To start the reaction, 50 µl Oxaloacetate (3 mM) were added, and the absorbance change at 412 nm was measured for 10 min at 37°C. The CS activity was calculated from the slope of the linear portion and normalized to mg tissue.

### Gene expression analysis

RNA isolation and quantitative real-time PCR (qPCR) was performed as described before [5]. *Quadriceps* muscle was used for analysis of SM gene expression.

### Histology

SM was fixed in 4% formaldehyde, embedded in paraffin and cut into 2 µm slices. Hematoxylin-eosin (H&E) staining (Roth, Fluka) was performed to visualize nuclear and cytoplasmic sections within the cell. Quantification of cross-sectional area was performed using Adiposoft (1.0) (Center for Applied Medical Research (CIMA) of the University of Navarra, Spain) [39].

### Western blot analysis

Protein was prepared from frozen skeletal muscle (*Quadriceps* muscle). Protein isolation, immunoblotting and detection were performed as previously described [5]. Immunoblots were performed using primary antibodies from Abcam against PGC-1α+β (#ab72230) and UCP1 (uncoupling protein 1, #ab23841); from BD Biosciences against CaMK-IV (Ca2+/calmodulin-dependent protein kinase IV), #610275); from Cell Signaling against ACC (#3662), AMPKα (AMP-activated protein kinase alpha, #2603), p-AMPKα (#2531), ATF4 (activating transcription factor 4, #11815), p-eIF2α (phospho-eukaryotic initiation factor 2 alpha, #3597), eIF2α (#5324), p-ERK1/2 (phospho-p44/42 MAPK, #4377), ERK1/2 (p44/42 MAPK, #4695), GLUT4 (glucose transporter 4, #2213), Hexokinase II (#2106), MFN2 (Mitofusin-2, #9482), p-Raptor (#2083), Raptor (#2280), p-ULK1 (#5869), ULK1 (#8054); from Enzo Life Sciences against HSP25 (heat shock protein 25, #ADI-SPA-801) and HSP70 (#ADI-SPA-810); from Gentaur against c-MYC (#04-CMYC-9E10); from Millipore against AMPKα1 (#07-350); from Progen against P62 (SQSTM1, #GP62-C); from MitoSciences against OXPHOS (MitoProfile total OXPHOS protein, #MS604); from R&D Systems against CD36 (#MAB2519), FGF21 (Fibroblast growth factor 21, #AF3057); from Santa Cruz against p-ACC2 (#sc-30446-R), AMPKα2 (sc-19131); and against GLUT1 (glucose transporter 1, kindly provided by Prof. Annette Schuermann (Department of Experimental Diabetology, DIfE, Potsdam-Rehbruecke, Germany). Protein expression was normalized to MFN2 which was not differentially expressed either on gene or on protein level between the 4 groups. Expression of the most frequently used housekeeper, the cytoskeleton protein α-Tubulin, was significantly increased in analyzed *Quadriceps* muscles of DTG mice (data not shown), a phenomenon also discussed for other experimental models with muscular dysfunction [40], [41].

### Skeletal muscle mitochondrial respiration

*Soleus* muscles were rapidly excised from isoflurane anesthetized mice and immediately placed in ice-cold biopsy preservation medium processed as described here [42]. Briefly, muscle samples were gently dissected with a pair of fine-tipped forceps and separated fibers were permeabilized with saponin (50 µg/ml) for 30 min at 4°C [43]. Muscle fibers were washed with mitochondrial respiration medium (MiR05, pH 7.1) containing 0.5 mM EGTA, 3 mM MgCl_2_•6H_2_O, 60 mM K-lactobionate, 20 mM Taurine, 10 mM KH_2_PO_4_, 20 mM HEPES, 110 mM Sucrose, 1 g/l BSA (fatty acid free) for 10 min at 4°C. Oxygen consumption was quantified polarographically at 37°C using the high-resolution Oxygraph-2k (OROBOROS Instruments, Innsbruck, Austria). For normalization of oxygen flux (O_2_/s*mg fiber), muscle wet weight of dry blotted fiber bundles was determined (AT200 scale, METTLER TOLEDO, Switzerland) prior to every tracing. Basal state 2 respiration of fibers in the absence of adenylates (ADP) was induced with the addition of 2 mM malate (M) and 0.2 mM octanoyl carnitine (O). Subsequently, coupled state 3 respiration was determined stepwise by adding 5 mM ADP (MO, maximal fatty acid oxidative capacity), followed by 5 mM pyruvate and 10 mM glutamate (MOPG, submaximal state 3 via complex I) and 10 mM succinate (MOPGS, maximal state 3). As an internal control of the outer mitochondrial membrane integrity, 10 µM cytochrome c was added upon maximal coupled respiration (Data not shown) [44]. Finally, 0.5 µM of the chemical uncoupler FCCP was added to evaluate the maximal capacity of the electron transport chain (state U, uncoupled respiration). All experiments were carried out in a hyperoxygenated environment to prevent potential oxygen diffusion limitation [45].

### Data analysis

Statistical analyses were performed using Stat Graph Prism (5.0). Data are reported as mean ± SEM. 1way ANOVA and Bonferroni's multiple comparisons test was used to evaluate differences between the genotypes. Statistical differences between the genotypes are indicated by superscript letters. Means annotated with different letters are significantly different. Statistical significance was assumed at P<0.05.

## Results

### Phenotypic characterization of DTG mice

**Figure 1:**
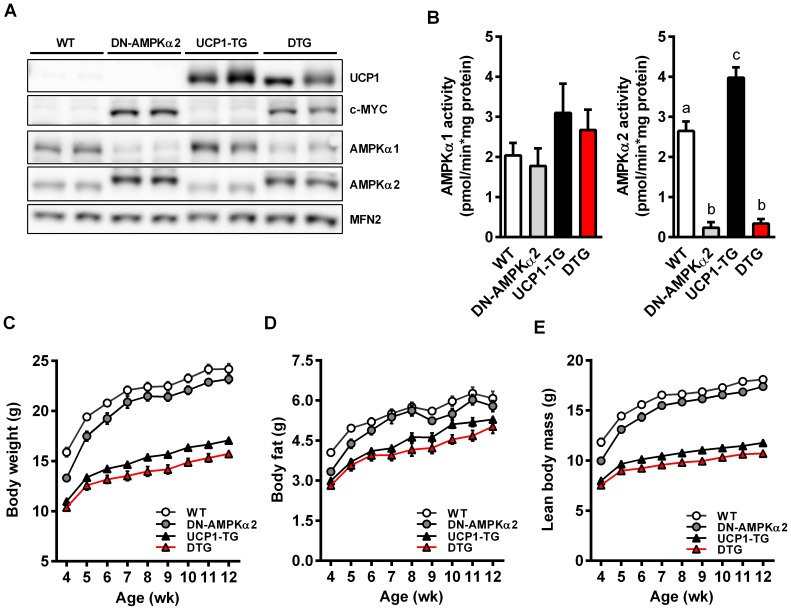
Phenotypic characterization of DTG mice. (A) Representative western blots of *Quadriceps* muscle from 12-wk-old WT, DN-AMPKα2, UCP1-TG and DTG mice, Mitofusin-2 (MFN2) was used as a loading control (n = 2 out of 6–8 analyzed per group). (B) Activity assay of catalytic AMPKα1 and AMPKα2 subunits in *Gastrocnemius* muscle from 12-wk-old WT, DN-AMPKα2, UCP1-TG and DTG mice (n = 5 per group). (C–E) Development of body weight and body composition from week 4 up to week 12 of age (n = 9–10 per goup). Data are the mean ± SEM. Means with different letters are significantly different (1way ANOVA and Bonferroni's multiple comparisons test, p<0.05).

Figure 1A shows protein expression of UCP1 and catalytic AMPK subunits in skeletal muscle of the 4 different genotypes. UCP1 was only detectable in UCP1-TG and DTG mice. The mutated inactive AMPKα2 contains a myc tag resulting in a higher molecular mass [29] evident in DN-AMPKα2 and DTG mice. Protein expression of AMPKα1, the second, minor isoform present in muscle is further decreased in DN-AMPKα2 and DTG mice, probably due to the increased abundance of the transgenic α2 subunit and the displacement of endogenous AMPKα1 from the βγ heterotrimer as previously suggested [29]. Published evidence for a UCP1 induced activation of AMPK in SM so far relies on the demonstration of AMPK phosphorylation only [4], [6]. Here we show for the first time that basal AMPKα2 activity is indeed significantly upregulated in SM muscle of UCP1-TG mice (Figure 1B). Expression of the kinase dead AMPKα2 almost completely abolished AMPKα2 activity in SM of DN-AMPKα2 as well as DTG mice. Importantly, AMPKα1 activity was not affected in muscle of DN-AMPKα2 and DTG mice (Figure 1B), revealing no compensatory induction of AMPKα1 as observed in SM of AMPKα2 whole-body knockout mice [46]. As previously shown, UCP1-TG mice were significantly smaller than WT as evident by a decreased body length, lean mass, fat mass and thus decreased total body weight (Figure 1C–E, table 1). This was preserved in DTG mice which even had a significantly reduced body length compared to all other groups (table 1) which was most likely due to a severe kyphosis observed in DTG mice (data not shown). DN-AMPKα2 had no major effects on body size, body composition, and energy balance (Figure 1C–E, table 1). Interestingly, due to the decreased relative lean and muscle mass, % body fat was greater in UCP1-TG and DTG mice than in WT and DN-AMPKα2 mice (table 1). Weight specific energy intake and expenditure were increased to the same extent in UCP1-TG and DTG mice compared to WT and DN-AMPKα2 mice (table 1). Moreover, the similar reduction of body weight in UCP1-TG and DTG mice compared to WT and DN-AMPKα2 mice under standard chow diet was preserved up to 35 weeks of age (Figure S1).

**Table 1:** Biometrical data and energy balance*.

	WT	DN-AMPKα2	UCP1-TG	DTG	p-value
Body weight (g)	24.2±0.5^a^	22.9±0.4^a^	16.7±0.2^b^	15.3±0.4^b^	p<0.0001
% body fat	25.9±0.5^a^	26.3±0.5^a^	31.1±0.7^b^	30.5±0.8^b^	p<0.0001
% lean mass	74.2±0.5^a^	73.7±0.5^a^	68.9±0.7^b^	69.5±0.8^b^	p<0.0001
Body length (cm)	10.0±0.1^a^	9.9±0.1^a^	9.4±0.1^b^	8.8±0.1^c^	p<0.0001
Energy intake (kJ/g/d)	3.4 ±0.1^a^	3.5±0.1^a^	4.2±0.1^b^	4.2±0.1^b^	p<0.0001
Energy expenditure (kJ/g/d)	1.8±0.02^a^	1.8±0.04^a^	2.0±0.02^b^	2.2±0.06^b^	p<0.0001
Quadriceps muscle weight (mg)	363.1±9.3^a^	340.8±11.6^a^	119.2±4.9^b^	119.1±5.1^b^	p<0.0001
Blood glucose (mmol/l)	6.0±0.3^a^	7.6±0.3^b^	6.6±0.2^a,b^	6.8±0.3^a,b^	p<0.01
Insulin (µg/l)	0.3±0.02^a^	0.4±0.03^a^	0.2±0.02^b^	0.2±0.02^b^	p<0.01

*All data are from week 11 except body length and muscle weight which were measured in week 12 after sacrificing mice. n = 9–10. Means with different letters within one row are significantly different (1way ANOVA and Bonferroni's multiple comparisons test, p<0.05).

### Improved systemic glucose homeostasis independent of AMPKα2 activation

**Figure 2:**
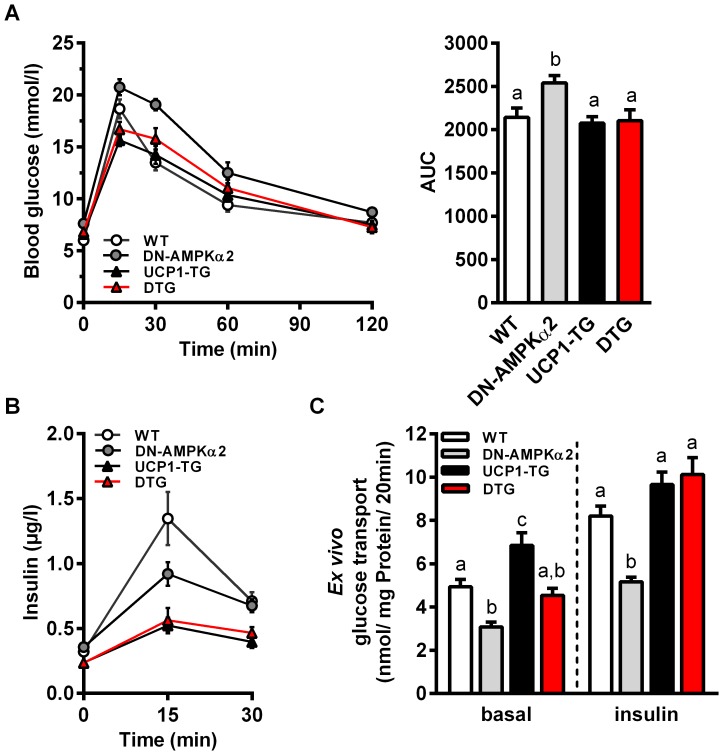
Improved systemic glucose homeostasis is independent of AMPKα2 activation. (A) Blood glucose levels, area under curve (AUC) of blood glucose and (B) plasma insulin levels during an oral glucose tolerance test (oGTT) in 10-wk-old WT, DN-AMPKα2, UCP1-TG and DTG mice (n = 9–10 per group). (C) *Ex vivo* basal and insulin-stimulated glucose uptake in intact *Extensor digitorum longus* (EDL) muscle from 12- to 17-wk-old WT, DN-AMPKα2, UCP1-TG and DTG mice (n = 10–15 per group). Data are the mean ± SEM. Means with different letters are significantly different (1way ANOVA and Bonferroni's multiple comparisons test, p<0.05).

Previously we have shown a diet independent increase in insulin sensitivity [2] and an increased basal and insulin activated glucose uptake in skeletal muscle from UCP1-TG mice [4]. UCP1-TG and DTG mice showed unchanged blood glucose levels but decreased plasma insulin compared to WT (table 1). For more detailed elucidation of glucose homeostasis we performed an oral glucose tolerance test (oGTT) and determined *ex vivo* glucose uptake into glycolytic EDL muscles as shown in Figure 2. Consistent with the finding that impairment of AMPKα2 activation affects SM glucose uptake [29], we observed slightly increased blood glucose levels (table 1) and reduced glucose tolerance (Figure 2A) in DN-AMPKα2 mice. All other genotypes showed very similar development of plasma glucose after glucose administration (Figure 2A). Notably, both UCP1-TG and DTG mice showed the same significantly reduced insulin levels compared to WT (Figure 2B). Together, these data suggest that positive effects of SM uncoupling on systemic glucose homeostasis occur independent of AMPKα2 activation. Exploration of glucose uptake in SM muscle itself showed, as expected, reduced basal and insulin stimulated *ex vivo* glucose uptake in DN-AMPKα2 mice (Figure 2C). UCP1-TG mice showed an increased basal glucose uptake compared to all other genotypes in line with increased gene expression of the basal glucose transporter GLUT1 (data not shown). Interestingly, the uncoupling induced increase in basal glucose uptake was completely abolished in SM of DTG mice, whereas the presence of UCP1 restored insulin induced glucose uptake in AMPKα2 ablated mice. In general, glucose uptake in SM is dpendent on glucose transporters, mainly GLUT4 and GLUT1, as well as on intracellular phosphorylation through hexokinase II [47]. However, Western blot analysis of glucose transporters showed no effect on GLUT1 protein levels but an increased GLUT4 protein expression in UCP1-TG which was curiously even higher in DTG mice (Figure S2). In addition, analysis of hexokinase II protein expression showed no differences between the genotypes (Figure S2).

### ACC phosphorylation and induction of CD36 is maintained in DTG mice

**Figure 3:**
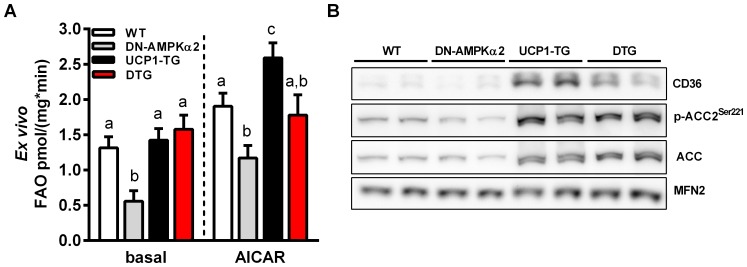
Fatty acid utilization is not disturbed in DTG mice. (A) *Ex vivo* basal and AICAR-stimulated fatty acid (FAO, [^3^H]-palmitate) oxidation in isolated intact *Soleus* muscle from 12- to 17-wk-old WT, DN-AMPKα2, UCP1-TG and DTG mice (n = 10-15 per group). (B) Representative western blots of *Quadriceps* muscle from 12-wk-old WT, DN-AMPKα2, UCP1-TG and DTG mice, Mitofusin-2 (MFN2) was used as a loading control (n = 2 out of 6–8 analyzed per group). Means with different letters are significantly different (1way ANOVA and Bonferroni's multiple comparisons test, p<0.05).

It has been shown that fatty acid oxidation is upregulated in SM of UCP1-TG mice [5] but only little is known about basal fatty acid metabolism in the DN-AMPKα2 mouse model. AMPK is thought to inhibit fatty acid synthesis and induce fatty acid oxidation through direct phosphorylation of the metabolic enzyme ACC2 [48], [49]. Controversially, recent published data suggest that AMPKα2 activation is not necessary for skeletal muscle fatty acid oxidation [50], [51]. As shown in Figure 3A, we found basal as well as AICAR stimulated fatty acid (palmitate) oxidation to be largely reduced in isolated SM of DN-AMPKα2 mice whereas UCP1-TG muscle showed increased AICAR stimulated but not basal fatty acid oxidation. This AICAR stimulated increase was abolished in DTG. On the other hand, UCP1 expression in DTG mice restored basal fatty oxidation to WT levels (Figure 3A). This was associated with an UCP1 induced, AMPKα2 independent increase in protein expression of CD36 (Figure 3B), a fatty acid transporter thought to be involved in cellular lipid oxidation [52], [53]. However, gene expression of CPT1B, a marker of mitochondrial fatty acid uptake was not different between the genotypes (data not shown). We further examined the phosphorylation of ACC2, a known downstream target of AMPK. Whereas phosphorylation tended to be lower in DN-AMPKα2 mice, we detected increased phosphorylation in UCP1-TG muscle as shown previously [5] which was preserved in DTG mice (Figure 3B).

### Impaired activity tolerance and skeletal muscle morphology in DTG mice

**Figure 4:**
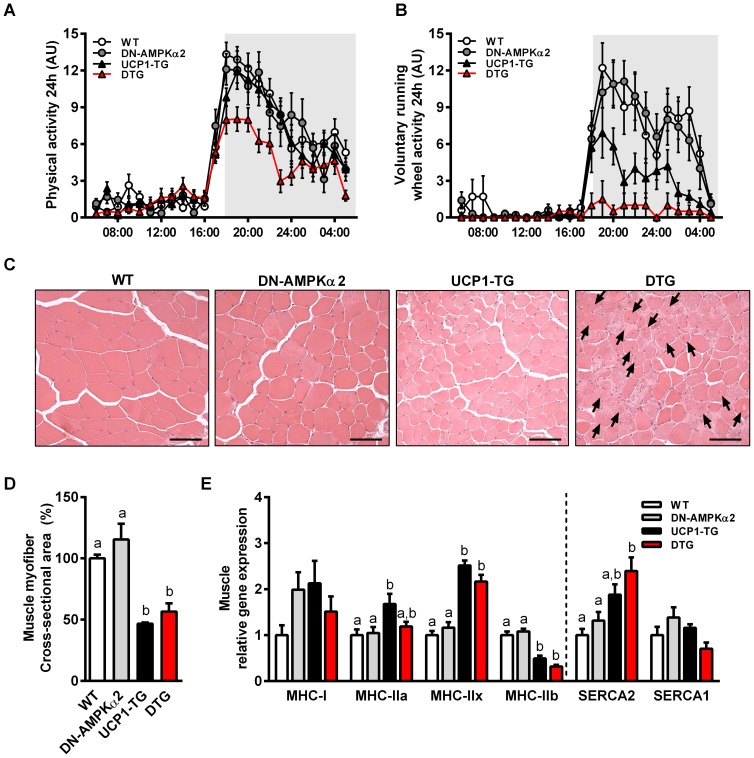
Impaired activity tolerance and skeletal muscle morphology in DTG mice. (A) Cage activity and (B) voluntary running wheel activity over 24 hrs of 11-wk-old WT, DN-AMPKα2, UCP1-TG and DTG mice (n = 6–10 per group). (C) Representative H&E staining of *M. tibialis anterior* (TA) muscle from 20-wk-old WT, DN-AMPKα2, UCP1-TG and DTG mice showing progressive muscle degeneration, including central nuclei (black arrows) in DTG mice, (scale bars 50 µm). (D) Quantification of cross-sectional area (CSA) of myofibers (n = 3 per group). (E) Gene expression analysis of muscle fiber-type markers in *Quadriceps* muscle of 12-wk-old WT, DN-AMPKα2, UCP1-TG and DTG mice by quantitative RT-PCR (n = 8 per group). Means with different letters are significantly different (1way ANOVA and Bonferroni's multiple comparisons test, p<0.05).

In order to assess muscle function and integrity we measured physical activity and performed SM histology. Both overall cage activity and voluntary running wheel activity were reduced in DTG mice (Figure 4A+B, Figure S3A). Remarkably, running wheel activity was almost absent in DTG mice, suggesting a severely compromised exercise capacity. Contrary to previous observations [29], we did not observe any differences in locomotor activity in DN-AMPKα2 mice compared to wildtype, whereas UCP1-TG mice showed slightly, but not significantly reduced running wheel activity (Figure 4B, Figure S3A). Overall skeletal muscle morphology was not affected in DN-AMPKα2 and UCP1-TG mice, although the latter as well as DTG mice displayed significantly smaller muscle fiber bundles (Figure 4C+D) consistent with reduced lean and muscle mass already observable at young age of 4 weeks (table 1, Figure S3D). Strikingly, DTG muscles showed severe degenerative changes in SM morphology, characterized by approximately 20% centronuclear myofibers (Figure 4C, Figure S3B). However, morphological changes in DTG muscle where independent of myogenin (MYOG) induction, a cellular marker indicative of myogenesis and regenerative processes in muscle [54], which was increased in both UCP1-TG and DTG mice (Figure S3C). Taken together, these data demonstrate the onset of a severe muscle dysfunction when UCP1 expression is combined with ablation of AMPKα2 activity. In addition, it has been reported that UCP1 expression in SM leads to a fast to slow fiber type switch by decreasing type IIb and increasing type IIa and IIx fiber content [55] which was confirmed here in *Quadriceps* muscle (Figure 4E). Except of an increased MHC-IIa expression, these changes in muscle fiber type markers were mainly preserved in DTG whereas DN-AMPKα2 mice did not show any differences in muscle fiber type compared to WT (Figure 4E). This suggests that AMPKα2 activation is not necessary for the mitochondrial uncoupling induced fiber type shift towards more oxidative fibers.

### Increased cellular stress response and suppressed AMPKα2-dependent induction of mitophagy regulator ULK1 in DTG mice

**Figure 5:**
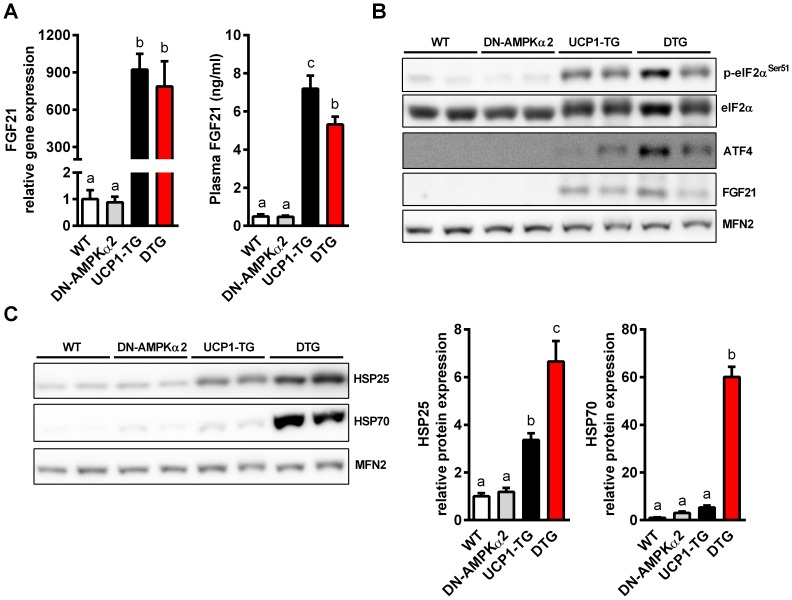
Increased cellular stress response in muscle of DTG mice. (A) Gene expression analysis of FGF21 in *Quadriceps* muscle by quantitative RT-PCR (n = 8 per group) and (B) FGF21 plasma concentration of 12-wk-old WT, DN-AMPKα2, UCP1-TG and DTG mice (n = 9–10 per group). (B) Representative western blots of proteins involved in integrated stress response and FGF21 induction and (C) of cellular stress response markers HSP25 and HSP70 in *Quadriceps* muscle from 12-wk-old WT, DN-AMPKα2, UCP1-TG and DTG mice, Mitofusin-2 (MFN2) was used as a loading control (n = 2 out of 6–8 analyzed per group). Means with different letters are significantly different (1way ANOVA and Bonferroni's multiple comparisons test, p<0.05).

Assessment of the gene expression profile of different AMPK and stress-related myokines [18], [56], [57] showed a significantly decreased expression of exercise-related FNDC5/irisin in line with the decreased physical activity of DTG mice (Figure S4). Moreover, gene expression of vascular endothelial growth factor beta (VEGF), an important AMPKα2-dependent regulator of basal capillary maintenance [18], was significantly decreased in DTG mice whereas IL15 and IL6 gene expression was not affected (Figure S4). We recently demonstrated that UCP1 expression in SM leads to an activation of the integrated stress response (ISR) evident by activation of the eukaryotic initiation factor 2 alpha (p-eIF2α)/activating transcription factor 4 (ATF4) cascade which in turn induces FGF21 gene expression and secretion [28]. As shown in Figure 5A, FGF21 gene and protein expression as well as circulating FGF21 in plasma was increased to the same extent in SM of UCP1-TG and DTG mice (Figure 5A+B). Furthermore, ISR induction was the same in UCP1-TG and DTG mice as evidenced by increased levels of p-eIF2α and ATF4. This shows that AMPKα2 activation is not required for this stress induced FGF21 induction. To assess markers of cellular stress status we measured heat shock protein (HSP) expression in SM. An increased expression of HSP25 and HSP70 in muscle of UCP1-TG was shown previously by our group [5]. This expression was even higher in muscles DTG mice (Figure 5C), indicating an elevated cellular stress level in SM of DTG mice.

We further measured AMPKα2-dependent regulators of SM autophagy. Phosphorylation of the unc-51-like kinase 1 (ULK1) by AMPK at Ser555 is critical for starvation-induced mitophagy, cell survival under conditions of low nutrients and energy, and mitochondrial homeostasis [58]. Mitophagy promoting AMPK-dependent phosphorylation of raptor and ULK1 was increased in SM of UCP1-TG mice (Figure S5), in line with our previous study showing induction of overall autophagy machinery in muscle UCP1-TG mice [28]. Strikingly, raptor and ULK1 phosphorylation was completely abolished in muscle of DN-AMPKα2 and DTG mice, likely due to the inactivation of AMPKα2 (Figure S5). However, the established marker for impaired autophagy, P62 [59], was not accumulated in muscles of DTG mice but rather decreased (Figure S5), indicating no general suppression of skeletal muscle autophagy flux.

### Reduced muscle mitochondrial function in DTG mice

**Figure 6:**
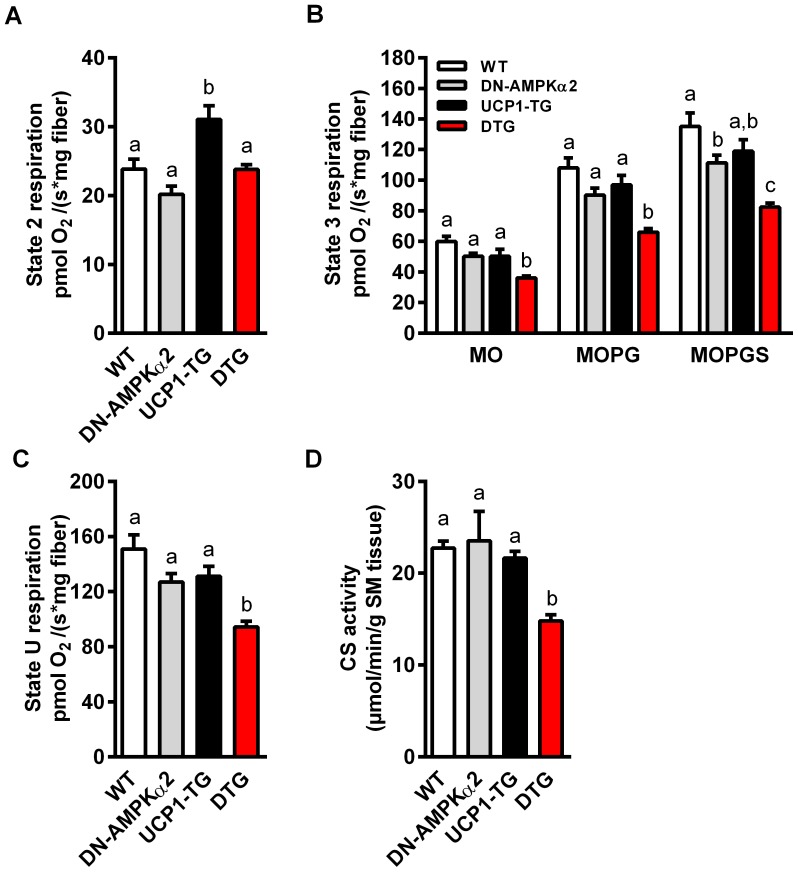
Diminished skeletal muscle mitochondrial respiratory capacity in DTG mice. (A–C) *Ex vivo* mitochondrial function assessed by measuring oxygen consumption of permeabilized muscle fibers of *Soleus* muscle from 17- to 23-wk-old WT, DN-AMPKα2, UCP1-TG and DTG mice; (A) upon substrates only (MO, malate + octanoylcarnitine, state 2 respiration), (B) ADP-stimulated respiration (state 3 respiration) fuelled by different substrates for complex I (MOPG, MO + pyruvate + glutamate) and complex II (MOPGS, MOPG + succinate), and (C) maximal uncoupled respiration (state U) after addition of the chemical uncoupler FCCP (n = 6–8 per group). (D) Citrate synthase (CS) activity in *Quadriceps* muscle of same mice as indicator of total mitochondrial capacity (n = 5–6 per group). Means with different letters are significantly different (1way ANOVA and Bonferroni's multiple comparisons test, p<0.05).

**Figure 7:**
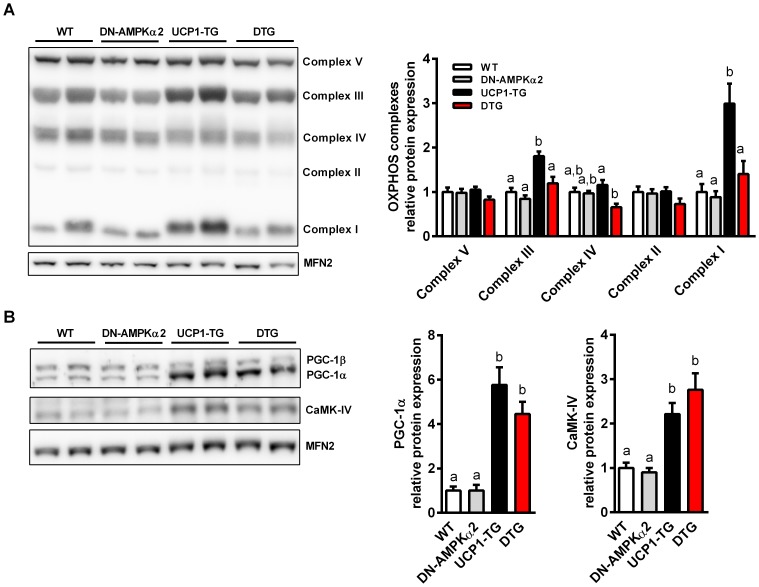
Suppressed mitochondrial OXPHOS induction in DTG mice. (A) Representative western blots and relative quantification of OXPHOS proteins and (B) of key regulators of mitochondrial biogenesis in *Quadriceps* muscle from WT, DN-AMPKα2, UCP1-TG and DTG mice. Mitofusin-2 (MFN2) was used as a loading control (n = 2 out of 6–8 analyzed per group). Means with different letters are significantly different (1way ANOVA and Bonferroni's multiple comparisons test, p<0.05).

Skeletal muscle integrity and performance is dependent on oxidative metabolism and mitochondrial function. Moreover, AMPK seems to be important for regulation of mitochondrial biogenesis in response to energy deprivation, particularly via two master regulators of mitochondrial biogenesis, peroxisome proliferator-activated receptor gamma coactivator 1-alpha (PGC-1α) and calcium/calmodulin-dependent protein kinase IV (CaMK-IV) [60], [61]. To assess whether the observed morphological differences in SM and the severe activity intolerance of DTG mice are due to a reduced mitochondrial function, we studied functional respiratory capacity of isolated permeabilized muscle fibers of *Soleus* muscle. Here we show that inactivation of AMPKα2 without any intervention or challenge had no effect on skeletal muscle fiber respiration of DN-AMPKα2 mice (Figure 6A–C). Furthermore, UCP1-TG showed no impairment of respiratory chain capacity (Figure 6B+C), and under state 2 conditions an even increased respiration (Figure 6A), due to the uncoupling via UCP1. On the other hand, muscle fibers of DTG mice overall showed significantly lower state 3 and uncoupled respiratory capacities (Figure 6B+C) in line with suppressed muscle citrate synthase activity (Figure 6D) and impaired SM morphology. Additionally, the Induction of OXPHOS protein expression in UCP1-TG muscle, also shown previously [5], was completely abolished in DTG mice (Figure 7A). Interestingly, protein expression of PGC-1α and CaMK-IV were significantly increased in both UCP1-TG and DTG mice (Figure 7B).

## Discussion

Recently we have demonstrated that SM mitochondrial uncoupling increases muscle substrate metabolism and longevity in mice [3], [5]. Moreover, improved glucose homeostasis, i.e. increased insulin sensitivity accompanied by decreased insulin levels and increased muscle glucose uptake is very robust metabolic phenotype of the UCP1-TG model. In addition, it was shown that UCP1 expression in skeletal muscle leads to an increased phosphorylation of AMPK [4], [6], thus linking the metabolic improvements of skeletal muscle uncoupling to an activated AMPK signaling.

Using double transgenic (DTG) mice with both SM UCP1 expression and AMPKα2 ablation, we show here that AMPKα2 is in fact dispensable for SM mitochondrial uncoupling induced beneficial metabolic effects on whole body energy balance, glucose homeostasis and basal fatty acid metabolism. This is in line with numerous studies reporting controversial data concerning the importance of AMPKα2 for regulation of glucose uptake and fatty acid oxidation, particularly when studied in SM [51], [62]–[64].

Inactivation of AMPKα2 in SM of young DTG mice had no effect on *in vivo* glucose tolerance or *ex vivo* maximum insulin-stimulated glucose transport of *EDL* muscle but abolished the uncoupling induced increase in basal glucose uptake. GLUT1 is thought to be primarily responsible for basal, constitutive glucose transport [65] but its muscle protein expression was similar in all four genotypes suggesting that GLUT1 overall abundance is not linked to differences in basal glucose transport. We observed an increased GLUT4 protein expression in muscle of UCP1-TG mice which was preserved or even higher in DTG mice. Additionally to glucose transporter expression, the expression of hexokinase II is thought to play an necessary role in regulation of glucose transport in muscle [66], whereas a possible regulation via AMPK activity is controversally discussed [16], [46]. However, we did not observe any differences in hexokinase II protein expression, excluding it's relevance here in UCP1-TG mice. Hence, further studies examining the molecular mechanisms responsible of basal glucose uptake in muscle of UCP1-TG mice are warranted.

Apart from potential regulation of glucose homeostasis, AMPK has been suggested as a key regulator of skeletal muscle fatty acid metabolism [48], [67]. Performing *ex vivo* analysis using oxidative *Soleus* muscles, we demonstrated that young DN-AMPKα2 mice had a highly suppressed basal and AICAR stimulated fatty acid (palmitate) oxidation. Strikingly, this was completely (basal) or partially (AICAR stimulated) recovered by increased mitochondrial uncoupling in DTG mice, despite a highly suppressed AMPKα2 activity and AMPKα1 protein expression as well as no compensatory increase in AMPKα1 activity. Other kinases have been suggested to play a role in skeletal muscle fatty acid uptake, such as the extra cellular signaling receptor kinase (ERK1/2) [68]. Expression and translocation of CD36 in rodent SM seems to require activation of ERK1/2 signaling [68]. We recently showed that ERK2 is activated in SM of UCP1-TG mice [5]. This increased phosphorylation of ERK2 was entirely preserved in DTG mice (Figure S6). However, Dzamko et al. demonstrated that ERK2 is not able to phosphorylate ACC2 at Ser221 [51] and further AMPK-independent acting kinases involved in muscle fatty acid oxidation are still not identified.

Generally, we cannot rule out completely that residual AMPKα2 or endogenous AMPKα1 activities in muscle of DTG mice are sufficient for the increased phosphorylation of ACC2, and for coordination of glucose homeostasis. However, we could not detect any compensatory increase of AMPKα1 protein expression or AMPKα1 activity in DTG mice, which were both described in AMPKα2 knockout mice [69]. Importantly, only AMPKα2 activity was highly induced in muscle of UCP1-TG mice, suggesting a higher relevance of the α2 subunit rather than α1 in response to chronic metabolic stress in SM.

Intriguing, fragility of DTG mice with reduced overall tonicity and observed muscle degenerative changes demonstrate that SM indeed highly suffered from reduction of AMPKα2 activity when exposed to chronic increase in metabolic demand. DTG mice behaved lazy and extremely activity intolerant, even in young age and under sedentary conditions. This could be interpreted as a compensatory behavior, where low AMPKα2 activity is sufficient to maintain whole body energy balance. This impairment of overall muscle function was already observable at week 4 up to week 12 of age simple by handling DTG mice, suggesting a rather early disturbance of muscle homeostasis and integrity. In order to assess whether there is a progressive decline of muscle function, time course analysis of muscle morphology and mitochondrial function would be highly supportive. However, we consider from our data presented here, that congenitally chronic metabolic stress leads to an early muscular dysfunction when activation of AMPKα2 is disturbed.

What might be the molecular mechanisms for the impaired muscle integrity? Interestingly, we found a massive induction of HSP70 exclusively in SM of DTG mice. Various perturbations such as exercise [70], muscle injury and regeneration [71] are related to an induction of HSP70 in skeletal muscle. Contrary, reduction of HSP70 abundance is associated with reduced muscle mass and force [72], [73]. Interestingly, DTG mice represent both phenotypes; (I) muscle injury/regeneration, reflected by increased centronuclear myofibers, and (II) a reduced muscle mass. Importantly, induction of HSP70 is also discussed as an adaptive response to severe depletion of ATP and increased cellular proteotoxic stress [74], suggesting that dysfunction of AMPKα2 in muscles of DTG mice does disturb cellular energy balance and integrity. Moreover, it has been proposed that SM capillarization is regulated by metabolic demands for maintaining cellular energy homeostasis [75], [76], which is also under basal control of AMPKα2 via induction of VEGF [18]. Strikingly, whereas UCP1-TG mice showed a slightly increased VEGF gene expression, in line with our recently published data [28], VEGF expression was significantly reduced in SM of DTG mice, suggesting a diminished angiogenesis in response to muscle respiratory dysfunction. It will be of future interest to delineate the role of VEGF-mediated muscle capillary density and the relevance for regulating SM plasticity under conditions of increased chronic metabolic stress.

Despite severe morphological changes in SM of DTG mice and reduced AMPKα2 activity there was no evidence for a general inhibition of autophagy in muscle of DTG mice. This is likely due to the activation of other major autophagy inducing signaling cascades, such as the ER-stress/eIF2α pathway [77] or the mitogen-activated protein kinases (MAPK), including JNK1 [78] which we previously found to be activated in UCP1-TG mice [5]. However, it is suggested that AMPK is involved in the specific turnover of mitochondria through regulation of mitophagy, a special form of autophagy [8]. A major signaling pathway in AMPK-induced skeletal muscle mitophagy concerns the activation of the ULK1, which promotes the initial steps of mitophagy induction [79], [80], thus triggering that damaged or defective mitochondria are engulfed and degraded by autophagosomes, and their contents recycled for re-use [81]–[83]. Strikingly, increased ULK1 induction in muscle of UCP1-TG mice was completely abolished in SM of DN-AMPKα2 and DTG mice, suggesting that this is dependent on AMPKα2. Again, suppression of AMPK-dependent mitophagy might be dispensable for young healthy DN-AMPKα2 mice housed under sedentary conditions, whereas it should be much more detrimental for DTG mice which are exposed to a chronic cellular stress response. Among the signaling pathways of mitophagy induction in UCP1-TG mice, AMPKα2 thus could play a role as housekeeper for cellular or rather mitochondrial quality control to maintain energy homeostasis and cell survival following starvation, which should be addressed in future studies.

Finally, skeletal muscle integrity and performance is dependent on mitochondrial oxidative metabolism and function. Without any metabolic challenge, young DN-AMPKα2 mice showed no impairment of muscle morphology or mitochondrial respiratory function and content, as already proposed in the literature [69], [84]. Strikingly, we show here that total mitochondrial respiratory capacity measured *ex vivo* in isolated skeletal muscle fibers was severely decreased in DTG mice, but not in DN-AMPKα2 or UCP1-TG mice. Moreover, mitochondrial OXPHOS protein content was impaired in DTG mice, suggesting a detrimental outcome of AMPKα2 inactivation in respiratory uncoupled muscle. Interestingly, reduction of mitochondrial respiratory capacity and OXPHOS protein content in DTG mice occurred despite an induction of both master regulators CaMK-IV and PGC-1α. Regarding the mitochondrial uncoupling induced PGC-1α activity this suggests that AMPK-mediated posttranslational modifications, such as phosphorylation or deacetylation are important [14], [85], as already shown previously in response to SM respiratory uncoupling [6]. Although there are additional factors that appear to regulate PGC-1α activation such as the p38 MAPK pathway [86], further studies determining the mechanisms mediating this effect are required.

## Conclusion

In this study, we found that AMPKα2 is dispensable for SM mitochondrial uncoupling induced beneficial effects on whole body energy balance, glucose homeostasis and insulin sensitivity. Very recently we reported that many of the beneficial whole body metabolic effects of SM uncoupling can be linked to increased secretion of endocrine acting fibroblast growth factor 21 (FGF21) from skeletal muscle of UCP1-TG mice [28]. This is induced through an activation of the integrated stress response [25], [28] apparently independent of AMPKα2 activation as shown here. However, our data demonstrated that activation of AMPKα2 participate in the fine tuning of cellular adaptation to chronic metabolic stress in muscle particularly by maintaining mitochondrial respiratory capacity and cellular homeostasis. Overall, the orchestration of SM energy metabolism and metabolic improvements in UCP1-TG mice seems not to be dependent on a single master switch such as AMPKα2, but rather it consists of a complex not yet fully understood signaling network which precisely senses and integrates energy demand, substrate oxidation and cellular stress resistance of skeletal muscle.

## Supporting Information

Figure S1 **Similar body weight development of UCP1-TG and DTG mice during aging.** n = 3–16 per group. Data are the mean ± SEM. Means with different letters are significantly different (1way ANOVA and Bonferroni's multiple comparisons test, p<0.05).
(TIF)

Figure S2 **Protein expression of glucose transporters and hexokinase II (HK II) in SM of UCP1-TG and DTG mice.** Representative western blots and relative quantification of *Quadriceps* muscle from WT, DN-AMPKα2, UCP1-TG and DTG mice, Mitofusin-2 (MFN2) was used as a loading control (n = 2 out of 6-8 analyzed per group). Means with different letters are significantly different (1way ANOVA and Bonferroni's multiple comparisons test, p<0.05).
(TIF)

Figure S3 **Characterization of in muscle function, myofiber morphology, myogenesis and early lean mass development.** (A) Quantification of locomotor activity during night at 11 wks of age (n = 6–10 per group). (B) Percentage of centronuclear myofibers of *M. tibialis anterior* (TA) muscle from a 20-wk-old WT, a DN-AMPKα2, a UCP1-TG and a DTG mouse (n = 3 per group). (C) Gene expression analysis of myogenesis marker MYOG in *Quadriceps* muscle of 12-wk-old WT, DN-AMPKα2, UCP1-TG and DTG mice by quantitative RT-PCR (n = 8 per group). (D) Means with different letters are significantly different (1way ANOVA and Bonferroni's multiple comparisons test, p<0.05).
(TIF)

Figure S4 **Decreased FNDC5 (irisin) and VEGFB myokine expression in SM of DTG mice.** Gene expression analysis of selected myokines in *Quadriceps* muscle of 12-wk-old WT, DN-AMPKα2, UCP1-TG and DTG mice by quantitative RT-PCR (n = 8 per group). Means with different letters are significantly different (1way ANOVA and Bonferroni's multiple comparisons test, p<0.05).
(TIF)

Figure S5 **Suppressed induction of AMPK downstream targets important for mitophagy regulation in SM of DTG mice.** Representative western blot showing expression of key proteins (and AMPK targets) involved in mitophagy regulation in *Quadriceps* muscle from 12-wk-old WT, DN-AMPKα2, UCP1-TG and DTG mice, Mitofusin-2 (MFN2) was used as a loading control (n = 2 out of 6-8 analyzed per group).
(TIF)

Figure S6 **Activation ERK2 signaling in SM of UCP1-TG and DTG mice.** Representative western blot of *Quadriceps* muscle from 12-wk-old WT, DN-AMPKα2, UCP1-TG and DTG mice, Mitofusin-2 (MFN2) was used as a loading control (n = 2 out of 6-8 analyzed per group).
(TIF)

## References

[1] Corpeleijn E, Saris WH, Blaak EE (2009) Metabolic flexibility in the development of insulin resistance and type 2 diabetes: effects of lifestyle. Obes Rev 10: 178–193. 19207879 10.1111/j.1467-789X.2008.00544.x

[2] Katterle Y, Keipert S, Hof J, Klaus S (2008) Dissociation of obesity and insulin resistance in transgenic mice with skeletal muscle expression of uncoupling protein 1. Physiol Genomics 32: 352–359. 18042832 10.1152/physiolgenomics.00194.2007

[3] Keipert S, Voigt A, Klaus S (2011) Dietary effects on body composition, glucose metabolism, and longevity are modulated by skeletal muscle mitochondrial uncoupling in mice. Aging Cell 10: 122–136. 21070590 10.1111/j.1474-9726.2010.00648.x PMC3042149

[4] Neschen S, Katterle Y, Richter J, Augustin R, Scherneck S, et al (2008) Uncoupling protein 1 expression in murine skeletal muscle increases AMPK activation, glucose turnover, and insulin sensitivity in vivo. Physiol Genomics 33: 333–340. 18349383 10.1152/physiolgenomics.00226.2007

[5] Keipert S, Ost M, Chadt A, Voigt A, Ayala V, et al (2013) Skeletal muscle uncoupling-induced longevity in mice is linked to increased substrate metabolism and induction of the endogenous antioxidant defense system. Am J Physiol Endocrinol Metab 304: E495–506. 23277187 10.1152/ajpendo.00518.2012

[6] Gates AC, Bernal-Mizrachi C, Chinault SL, Feng C, Schneider JG, et al (2007) Respiratory uncoupling in skeletal muscle delays death and diminishes age-related disease. Cell Metab 6: 497–505. 18054318 10.1016/j.cmet.2007.10.010

[7] Klaus S, Keipert S, Rossmeisl M, Kopecky J (2011) Augmenting energy expenditure by mitochondrial uncoupling: a role of AMP-activated protein kinase. Genes Nutr. 10.1007/s12263-011-0260-8 PMC3380193 22139637

[8] Hardie DG, Ross FA, Hawley SA (2012) AMPK: a nutrient and energy sensor that maintains energy homeostasis. Nat Rev Mol Cell Biol 13: 251–262. 22436748 10.1038/nrm3311 PMC5726489

[9] Zhang BB, Zhou G, Li C (2009) AMPK: an emerging drug target for diabetes and the metabolic syndrome. Cell Metab 9: 407–416. 19416711 10.1016/j.cmet.2009.03.012

[10] Hawley SA, Davison M, Woods A, Davies SP, Beri RK, et al (1996) Characterization of the AMP-activated protein kinase kinase from rat liver and identification of threonine 172 as the major site at which it phosphorylates AMP-activated protein kinase. J Biol Chem 271: 27879–27887. 8910387 10.1074/jbc.271.44.27879

[11] O'Neill HM (2013) AMPK and Exercise: Glucose Uptake and Insulin Sensitivity. Diabetes Metab J 37: 1–21. 23441028 10.4093/dmj.2013.37.1.1 PMC3579147

[12] Hardie DG (2008) AMPK: a key regulator of energy balance in the single cell and the whole organism. Int J Obes (Lond) 32 Suppl 4S7–12. 18719601 10.1038/ijo.2008.116

[13] McGee SL, Hargreaves M (2010) AMPK-mediated regulation of transcription in skeletal muscle. Clin Sci (Lond) 118: 507–518. 20088830 10.1042/CS20090533

[14] Canto C, Jiang LQ, Deshmukh AS, Mataki C, Coste A, et al (2010) Interdependence of AMPK and SIRT1 for metabolic adaptation to fasting and exercise in skeletal muscle. Cell Metab 11: 213–219. 20197054 10.1016/j.cmet.2010.02.006 PMC3616265

[15] Viollet B, Athea Y, Mounier R, Guigas B, Zarrinpashneh E, et al (2009) AMPK: Lessons from transgenic and knockout animals. Front Biosci 14: 19–44. 10.2741/3229 PMC2666987 19273052

[16] Rockl KS, Hirshman MF, Brandauer J, Fujii N, Witters LA, et al (2007) Skeletal muscle adaptation to exercise training: AMP-activated protein kinase mediates muscle fiber type shift. Diabetes 56: 2062–2069. 17513699 10.2337/db07-0255

[17] Geng T, Li P, Okutsu M, Yin X, Kwek J, et al (2010) PGC-1alpha plays a functional role in exercise-induced mitochondrial biogenesis and angiogenesis but not fiber-type transformation in mouse skeletal muscle. Am J Physiol Cell Physiol 298: C572–579. 20032509 10.1152/ajpcell.00481.2009 PMC3353735

[18] Zwetsloot KA, Westerkamp LM, Holmes BF, Gavin TP (2008) AMPK regulates basal skeletal muscle capillarization and VEGF expression, but is not necessary for the angiogenic response to exercise. J Physiol 586: 6021–6035. 18955383 10.1113/jphysiol.2008.159871 PMC2655430

[19] O'Neill HM, Maarbjerg SJ, Crane JD, Jeppesen J, Jorgensen SB, et al (2011) AMP-activated protein kinase (AMPK) beta1beta2 muscle null mice reveal an essential role for AMPK in maintaining mitochondrial content and glucose uptake during exercise. Proc Natl Acad Sci U S A 108: 16092–16097. 21896769 10.1073/pnas.1105062108 PMC3179037

[20] Thomson DM, Porter BB, Tall JH, Kim HJ, Barrow JR, et al (2007) Skeletal muscle and heart LKB1 deficiency causes decreased voluntary running and reduced muscle mitochondrial marker enzyme expression in mice. Am J Physiol Endocrinol Metab 292: E196–202. 16926377 10.1152/ajpendo.00366.2006

[21] Liang J, Shao SH, Xu ZX, Hennessy B, Ding Z, et al (2007) The energy sensing LKB1-AMPK pathway regulates p27(kip1) phosphorylation mediating the decision to enter autophagy or apoptosis. Nat Cell Biol 9: 218–224. 17237771 10.1038/ncb1537

[22] Salminen A, Kaarniranta K (2012) AMP-activated protein kinase (AMPK) controls the aging process via an integrated signaling network. Ageing Res Rev 11: 230–241. 22186033 10.1016/j.arr.2011.12.005

[23] Sanchez AM, Candau RB, Csibi A, Pagano AF, Raibon A, et al (2012) The role of AMP-activated protein kinase in the coordination of skeletal muscle turnover and energy homeostasis. Am J Physiol Cell Physiol 303: C475–485. 22700795 10.1152/ajpcell.00125.2012

[24] Kroemer G, Marino G, Levine B (2010) Autophagy and the integrated stress response. Mol Cell 40: 280–293. 20965422 10.1016/j.molcel.2010.09.023 PMC3127250

[25] Kim KH, Jeong YT, Oh H, Kim SH, Cho JM, et al (2013) Autophagy deficiency leads to protection from obesity and insulin resistance by inducing Fgf21 as a mitokine. Nat Med 19: 83–92. 23202295 10.1038/nm.3014

[26] Masiero E, Agatea L, Mammucari C, Blaauw B, Loro E, et al (2009) Autophagy is required to maintain muscle mass. Cell Metab 10: 507–515. 19945408 10.1016/j.cmet.2009.10.008

[27] Sanchez AM, Csibi A, Raibon A, Cornille K, Gay S, et al (2012) AMPK promotes skeletal muscle autophagy through activation of forkhead FoxO3a and interaction with Ulk1. J Cell Biochem 113: 695–710. 22006269 10.1002/jcb.23399

[28] Keipert S, Ost M, Johann K, Imber F, Jastroch M, et al.. (2013) Skeletal muscle mitochondrial uncoupling drives endocrine cross-talk through induction of FGF21 as a myokine. Am J Physiol Endocrinol Metab in press. 10.1152/ajpendo.00330.2013 24347058

[29] Mu J, Brozinick JT Jr, Valladares O, Bucan M, Birnbaum MJ (2001) A role for AMP-activated protein kinase in contraction- and hypoxia-regulated glucose transport in skeletal muscle. Mol Cell 7: 1085–1094. 11389854 10.1016/s1097-2765(01)00251-9

[30] Mu J, Barton ER, Birnbaum MJ (2003) Selective suppression of AMP-activated protein kinase in skeletal muscle: update on ‘lazy mice’. Biochem Soc Trans 31: 236–241. 12546693 10.1042/bst0310236

[31] Klaus S, Rudolph B, Dohrmann C, Wehr R (2005) Expression of uncoupling protein 1 in skeletal muscle decreases muscle energy efficiency and affects thermoregulation and substrate oxidation. Physiol Genomics 21: 193–200. 15687481 10.1152/physiolgenomics.00299.2004

[32] Keipert S, Klaus S, Heldmaier G, Jastroch M (2010) UCP1 ectopically expressed in murine muscle displays native function and mitigates mitochondrial superoxide production. Biochim Biophys Acta 1797: 324–330. 19958747 10.1016/j.bbabio.2009.11.008

[33] Davies SP, Carling D, Hardie DG (1989) Tissue distribution of the AMP-activated protein kinase, and lack of activation by cyclic-AMP-dependent protein kinase, studied using a specific and sensitive peptide assay. Eur J Biochem 186: 123–128. 2574667 10.1111/j.1432-1033.1989.tb15185.x

[34] Kudo N, Barr AJ, Barr RL, Desai S, Lopaschuk GD (1995) High rates of fatty acid oxidation during reperfusion of ischemic hearts are associated with a decrease in malonyl-CoA levels due to an increase in 5′-AMP-activated protein kinase inhibition of acetyl-CoA carboxylase. J Biol Chem 270: 17513–17520. 7615556 10.1074/jbc.270.29.17513

[35] Barnes BR, Marklund S, Steiler TL, Walter M, Hjalm G, et al (2004) The 5′-AMP-activated protein kinase gamma3 isoform has a key role in carbohydrate and lipid metabolism in glycolytic skeletal muscle. J Biol Chem 279: 38441–38447. 15247217 10.1074/jbc.M405533200

[36] Chadt A, Leicht K, Deshmukh A, Jiang LQ, Scherneck S, et al (2008) Tbc1d1 mutation in lean mouse strain confers leanness and protects from diet-induced obesity. Nat Genet 40: 1354–1359. 18931681 10.1038/ng.244

[37] Dokas J, Chadt A, Nolden T, Himmelbauer H, Zierath JR, et al (2013) Conventional knockout of Tbc1d1 in mice impairs insulin- and AICAR-stimulated glucose uptake in skeletal muscle. Endocrinology 154: 3502–3514. 23892475 10.1210/en.2012-2147

[38] Seebacher F, Guderley H, Elsey RM, Trosclair PL 3rd (2003) Seasonal acclimatisation of muscle metabolic enzymes in a reptile (Alligator mississippiensis). J Exp Biol 206: 1193–1200. 12604579 10.1242/jeb.00223

[39] Boque N, Campion J, Paternain L, Garcia-Diaz DF, Galarraga M, et al (2009) Influence of dietary macronutrient composition on adiposity and cellularity of different fat depots in Wistar rats. J Physiol Biochem 65: 387–395. 20358352 10.1007/BF03185934

[40] Mutsaers CA, Wishart TM, Lamont DJ, Riessland M, Schreml J, et al (2011) Reversible molecular pathology of skeletal muscle in spinal muscular atrophy. Hum Mol Genet 20: 4334–4344. 21840928 10.1093/hmg/ddr360

[41] Eaton SL, Roche SL, Llavero Hurtado M, Oldknow KJ, Farquharson C, et al (2013) Total protein analysis as a reliable loading control for quantitative fluorescent Western blotting. PLoS One 8: e72457. 24023619 10.1371/journal.pone.0072457 PMC3758299

[42] Jacobs RA, Diaz V, Meinild AK, Gassmann M, Lundby C (2013) The C57Bl/6 mouse serves as a suitable model of human skeletal muscle mitochondrial function. Exp Physiol 98: 908–921. 23180810 10.1113/expphysiol.2012.070037

[43] Kuznetsov AV, Schneeberger S, Seiler R, Brandacher G, Mark W, et al (2004) Mitochondrial defects and heterogeneous cytochrome c release after cardiac cold ischemia and reperfusion. Am J Physiol Heart Circ Physiol 286: H1633–1641. 14693685 10.1152/ajpheart.00701.2003

[44] Kuznetsov AV, Veksler V, Gellerich FN, Saks V, Margreiter R, et al (2008) Analysis of mitochondrial function in situ in permeabilized muscle fibers, tissues and cells. Nat Protoc 3: 965–976. 18536644 10.1038/nprot.2008.61

[45] Pesta D, Gnaiger E (2012) High-resolution respirometry: OXPHOS protocols for human cells and permeabilized fibers from small biopsies of human muscle. Methods Mol Biol 810: 25–58. 22057559 10.1007/978-1-61779-382-0_3

[46] Jorgensen SB, Treebak JT, Viollet B, Schjerling P, Vaulont S, et al (2007) Role of AMPKalpha2 in basal, training-, and AICAR-induced GLUT4, hexokinase II, and mitochondrial protein expression in mouse muscle. Am J Physiol Endocrinol Metab 292: E331–339. 16954334 10.1152/ajpendo.00243.2006

[47] Klip A, Paquet MR (1990) Glucose transport and glucose transporters in muscle and their metabolic regulation. Diabetes Care 13: 228–243. 2407478 10.2337/diacare.13.3.228

[48] Merrill GF, Kurth EJ, Hardie DG, Winder WW (1997) AICA riboside increases AMP-activated protein kinase, fatty acid oxidation, and glucose uptake in rat muscle. Am J Physiol 273: E1107–1112. 9435525 10.1152/ajpendo.1997.273.6.E1107

[49] Carling D, Clarke PR, Zammit VA, Hardie DG (1989) Purification and characterization of the AMP-activated protein kinase. Copurification of acetyl-CoA carboxylase kinase and 3-hydroxy-3-methylglutaryl-CoA reductase kinase activities. Eur J Biochem 186: 129–136. 2598924 10.1111/j.1432-1033.1989.tb15186.x

[50] Alkhateeb H, Holloway GP, Bonen A (2011) Skeletal muscle fatty acid oxidation is not directly associated with AMPK or ACC2 phosphorylation. Appl Physiol Nutr Metab 36: 361–367. 21574785 10.1139/h11-024

[51] Dzamko N, Schertzer JD, Ryall JG, Steel R, Macaulay SL, et al (2008) AMPK-independent pathways regulate skeletal muscle fatty acid oxidation. J Physiol 586: 5819–5831. 18845612 10.1113/jphysiol.2008.159814 PMC2655404

[52] Coburn CT, Knapp FF Jr, Febbraio M, Beets AL, Silverstein RL, et al (2000) Defective uptake and utilization of long chain fatty acids in muscle and adipose tissues of CD36 knockout mice. J Biol Chem 275: 32523–32529. 10913136 10.1074/jbc.M003826200

[53] Nahle Z, Hsieh M, Pietka T, Coburn CT, Grimaldi PA, et al (2008) CD36-dependent regulation of muscle FoxO1 and PDK4 in the PPAR delta/beta-mediated adaptation to metabolic stress. J Biol Chem 283: 14317–14326. 18308721 10.1074/jbc.M706478200 PMC2386936

[54] Sabourin LA, Girgis-Gabardo A, Seale P, Asakura A, Rudnicki MA (1999) Reduced differentiation potential of primary MyoD-/- myogenic cells derived from adult skeletal muscle. J Cell Biol 144: 631–643. 10037786 10.1083/jcb.144.4.631 PMC2132931

[55] Couplan E, Gelly C, Goubern M, Fleury C, Quesson B, et al (2002) High level of uncoupling protein 1 expression in muscle of transgenic mice selectively affects muscles at rest and decreases their IIb fiber content. J Biol Chem 277: 43079–43088. 12221093 10.1074/jbc.M206726200

[56] Bostrom P, Wu J, Jedrychowski MP, Korde A, Ye L, et al (2012) A PGC1-alpha-dependent myokine that drives brown-fat-like development of white fat and thermogenesis. Nature 481: 463–468. 22237023 10.1038/nature10777 PMC3522098

[57] Haddad F, Zaldivar F, Cooper DM, Adams GR (2005) IL-6-induced skeletal muscle atrophy. J Appl Physiol (1985) 98: 911–917. 15542570 10.1152/japplphysiol.01026.2004

[58] Egan DF, Shackelford DB, Mihaylova MM, Gelino S, Kohnz RA, et al (2011) Phosphorylation of ULK1 (hATG1) by AMP-activated protein kinase connects energy sensing to mitophagy. Science 331: 456–461. 21205641 10.1126/science.1196371 PMC3030664

[59] Bjorkoy G, Lamark T, Pankiv S, Overvatn A, Brech A, et al (2009) Monitoring autophagic degradation of p62/SQSTM1. Methods Enzymol 452: 181–197. 19200883 10.1016/S0076-6879(08)03612-4

[60] Zong H, Ren JM, Young LH, Pypaert M, Mu J, et al (2002) AMP kinase is required for mitochondrial biogenesis in skeletal muscle in response to chronic energy deprivation. Proc Natl Acad Sci U S A 99: 15983–15987. 12444247 10.1073/pnas.252625599 PMC138551

[61] Wu H, Kanatous SB, Thurmond FA, Gallardo T, Isotani E, et al (2002) Regulation of mitochondrial biogenesis in skeletal muscle by CaMK. Science 296: 349–352. 11951046 10.1126/science.1071163

[62] Holmes BF, Lang DB, Birnbaum MJ, Mu J, Dohm GL (2004) AMP kinase is not required for the GLUT4 response to exercise and denervation in skeletal muscle. Am J Physiol Endocrinol Metab 287: E739–743. 15165992 10.1152/ajpendo.00080.2004

[63] Jeppesen J, Albers PH, Rose AJ, Birk JB, Schjerling P, et al (2011) Contraction-induced skeletal muscle FAT/CD36 trafficking and FA uptake is AMPK independent. J Lipid Res 52: 699–711. 21297178 10.1194/jlr.M007138 PMC3053206

[64] Raney MA, Yee AJ, Todd MK, Turcotte LP (2005) AMPK activation is not critical in the regulation of muscle FA uptake and oxidation during low-intensity muscle contraction. Am J Physiol Endocrinol Metab 288: E592–598. 15547141 10.1152/ajpendo.00301.2004

[65] Olson AL, Pessin JE (1996) Structure, function, and regulation of the mammalian facilitative glucose transporter gene family. Annu Rev Nutr 16: 235–256. 8839927 10.1146/annurev.nu.16.070196.001315

[66] Fueger PT, Shearer J, Bracy DP, Posey KA, Pencek RR, et al (2005) Control of muscle glucose uptake: test of the rate-limiting step paradigm in conscious, unrestrained mice. J Physiol 562: 925–935. 15576451 10.1113/jphysiol.2004.076158 PMC1665542

[67] Thomson DM, Winder WW (2009) AMP-activated protein kinase control of fat metabolism in skeletal muscle. Acta Physiol (Oxf) 196: 147–154. 19245653 10.1111/j.1748-1716.2009.01973.x PMC2734509

[68] Turcotte LP, Raney MA, Todd MK (2005) ERK1/2 inhibition prevents contraction-induced increase in plasma membrane FAT/CD36 content and FA uptake in rodent muscle. Acta Physiol Scand 184: 131–139. 15916673 10.1111/j.1365-201X.2005.01445.x

[69] Viollet B, Athea Y, Mounier R, Guigas B, Zarrinpashneh E, et al (2009) AMPK: Lessons from transgenic and knockout animals. Front Biosci (Landmark Ed) 14: 19–44. 19273052 10.2741/3229 PMC2666987

[70] Milne KJ, Noble EG (2002) Exercise-induced elevation of HSP70 is intensity dependent. J Appl Physiol (1985) 93: 561–568. 12133865 10.1152/japplphysiol.00528.2001

[71] Senf SM, Howard TM, Ahn B, Ferreira LF, Judge AR (2013) Loss of the inducible Hsp70 delays the inflammatory response to skeletal muscle injury and severely impairs muscle regeneration. PLoS One 8: e62687. 23626847 10.1371/journal.pone.0062687 PMC3633856

[72] Senf SM, Dodd SL, McClung JM, Judge AR (2008) Hsp70 overexpression inhibits NF-kappaB and Foxo3a transcriptional activities and prevents skeletal muscle atrophy. FASEB J 22: 3836–3845. 18644837 10.1096/fj.08-110163 PMC6137947

[73] Lawler JM, Song W, Kwak HB (2006) Differential response of heat shock proteins to hindlimb unloading and reloading in the soleus. Muscle Nerve 33: 200–207. 16258950 10.1002/mus.20454

[74] Kabakov AE, Budagova KR, Latchman DS, Kampinga HH (2002) Stressful preconditioning and HSP70 overexpression attenuate proteotoxicity of cellular ATP depletion. Am J Physiol Cell Physiol 283: C521–534. 12107062 10.1152/ajpcell.00503.2001

[75] Adair TH (2005) Growth regulation of the vascular system: an emerging role for adenosine. Am J Physiol Regul Integr Comp Physiol 289: R283–R296. 16014444 10.1152/ajpregu.00840.2004

[76] Hepple RT, Mackinnon SL, Goodman JM, Thomas SG, Plyley MJ (1997) Resistance and aerobic training in older men: effects on VO2peak and the capillary supply to skeletal muscle. J Appl Physiol (1985) 82: 1305–1310. 9104869 10.1152/jappl.1997.82.4.1305

[77] Kouroku Y, Fujita E, Tanida I, Ueno T, Isoai A, et al (2007) ER stress (PERK/eIF2alpha phosphorylation) mediates the polyglutamine-induced LC3 conversion, an essential step for autophagy formation. Cell Death Differ 14: 230–239. 16794605 10.1038/sj.cdd.4401984

[78] Wong CH, Iskandar KB, Yadav SK, Hirpara JL, Loh T, et al (2010) Simultaneous induction of non-canonical autophagy and apoptosis in cancer cells by ROS-dependent ERK and JNK activation. PLoS One 5: e9996. 20368806 10.1371/journal.pone.0009996 PMC2848860

[79] Mizushima N (2010) The role of the Atg1/ULK1 complex in autophagy regulation. Curr Opin Cell Biol 22: 132–139. 20056399 10.1016/j.ceb.2009.12.004

[80] Lee JW, Park S, Takahashi Y, Wang HG (2010) The association of AMPK with ULK1 regulates autophagy. PLoS One 5: e15394. 21072212 10.1371/journal.pone.0015394 PMC2972217

[81] Egan D, Kim J, Shaw RJ, Guan KL (2011) The autophagy initiating kinase ULK1 is regulated via opposing phosphorylation by AMPK and mTOR. Autophagy 7: 643–644. 21460621 10.4161/auto.7.6.15123 PMC3359466

[82] Kim J, Kundu M, Viollet B, Guan KL (2011) AMPK and mTOR regulate autophagy through direct phosphorylation of Ulk1. Nat Cell Biol 13: 132–141. 21258367 10.1038/ncb2152 PMC3987946

[83] Jung CH, Jun CB, Ro SH, Kim YM, Otto NM, et al (2009) ULK-Atg13-FIP200 complexes mediate mTOR signaling to the autophagy machinery. Mol Biol Cell 20: 1992–2003. 19225151 10.1091/mbc.E08-12-1249 PMC2663920

[84] Jorgensen SB, Wojtaszewski JF, Viollet B, Andreelli F, Birk JB, et al (2005) Effects of alpha-AMPK knockout on exercise-induced gene activation in mouse skeletal muscle. FASEB J 19: 1146–1148. 15878932 10.1096/fj.04-3144fje

[85] Jager S, Handschin C, St-Pierre J, Spiegelman BM (2007) AMP-activated protein kinase (AMPK) action in skeletal muscle via direct phosphorylation of PGC-1alpha. Proc Natl Acad Sci U S A 104: 12017–12022. 17609368 10.1073/pnas.0705070104 PMC1924552

[86] Akimoto T, Pohnert SC, Li P, Zhang M, Gumbs C, et al (2005) Exercise stimulates Pgc-1alpha transcription in skeletal muscle through activation of the p38 MAPK pathway. J Biol Chem 280: 19587–19593. 15767263 10.1074/jbc.M408862200

