# Supplementary figures and images for: Activation of AMPKα2 Is Not Crucial for Mitochondrial Uncoupling-Induced Metabolic Effects but Required to Maintain Skeletal Muscle Integrity
Source: PLoS One. 2014 Apr 14;9(4):e94689. doi: 10.1371/journal.pone.0094689 (PMC3986237; doi:10.1371/journal.pone.0094689)

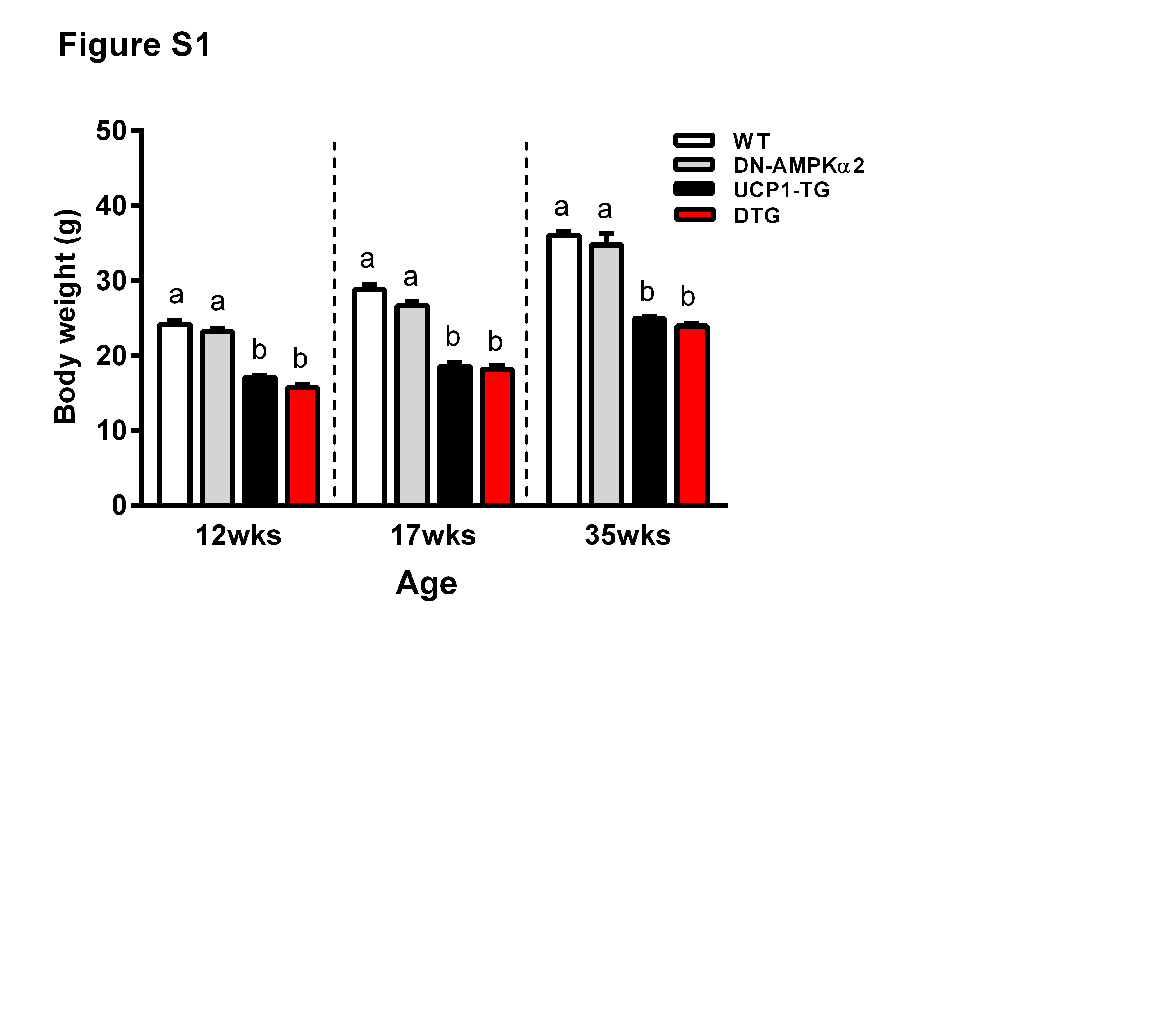

Supplement: Figure S1 — Similar body weight development of UCP1-TG and DTG mice during aging. n = 3–16 per group. Data are the mean ± SEM. Means with different letters are significantly different (1way ANOVA and Bonferroni's multiple comparisons test, p<0.05). (TIF) [file pone.0094689.s001.tif]

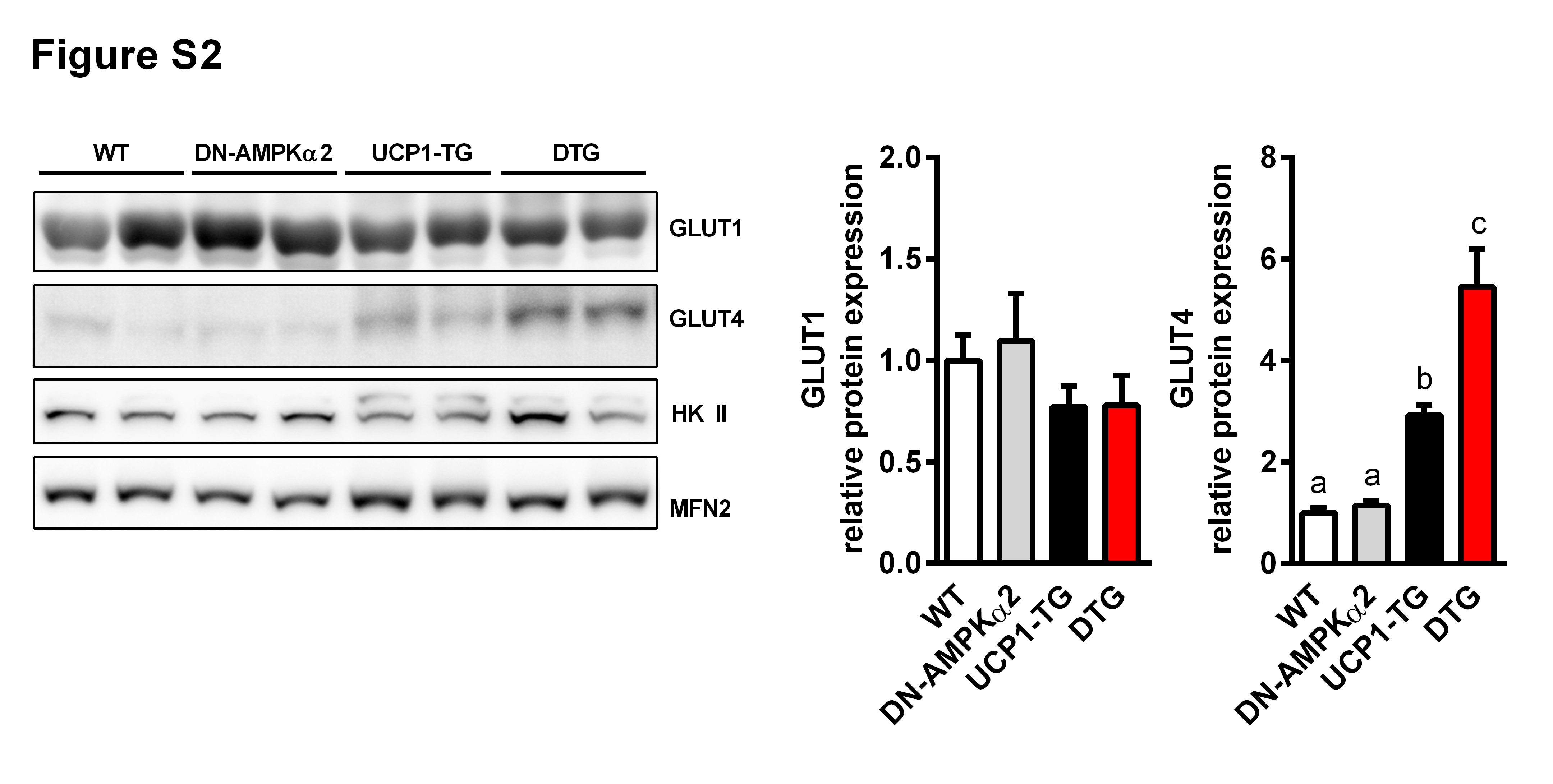

Supplement: Figure S2 — Protein expression of glucose transporters and hexokinase II (HK II) in SM of UCP1-TG and DTG mice. Representative western blots and relative quantification of Quadriceps muscle from WT, DN-AMPKα2, UCP1-TG and DTG mice, Mitofusin-2 (MFN2) was used as a loading control (n = 2 out of 6-8 analyzed per group). Means with different letters are significantly different (1way ANOVA and Bonferroni's multiple comparisons test, p<0.05). (TIF) [file pone.0094689.s002.tif]

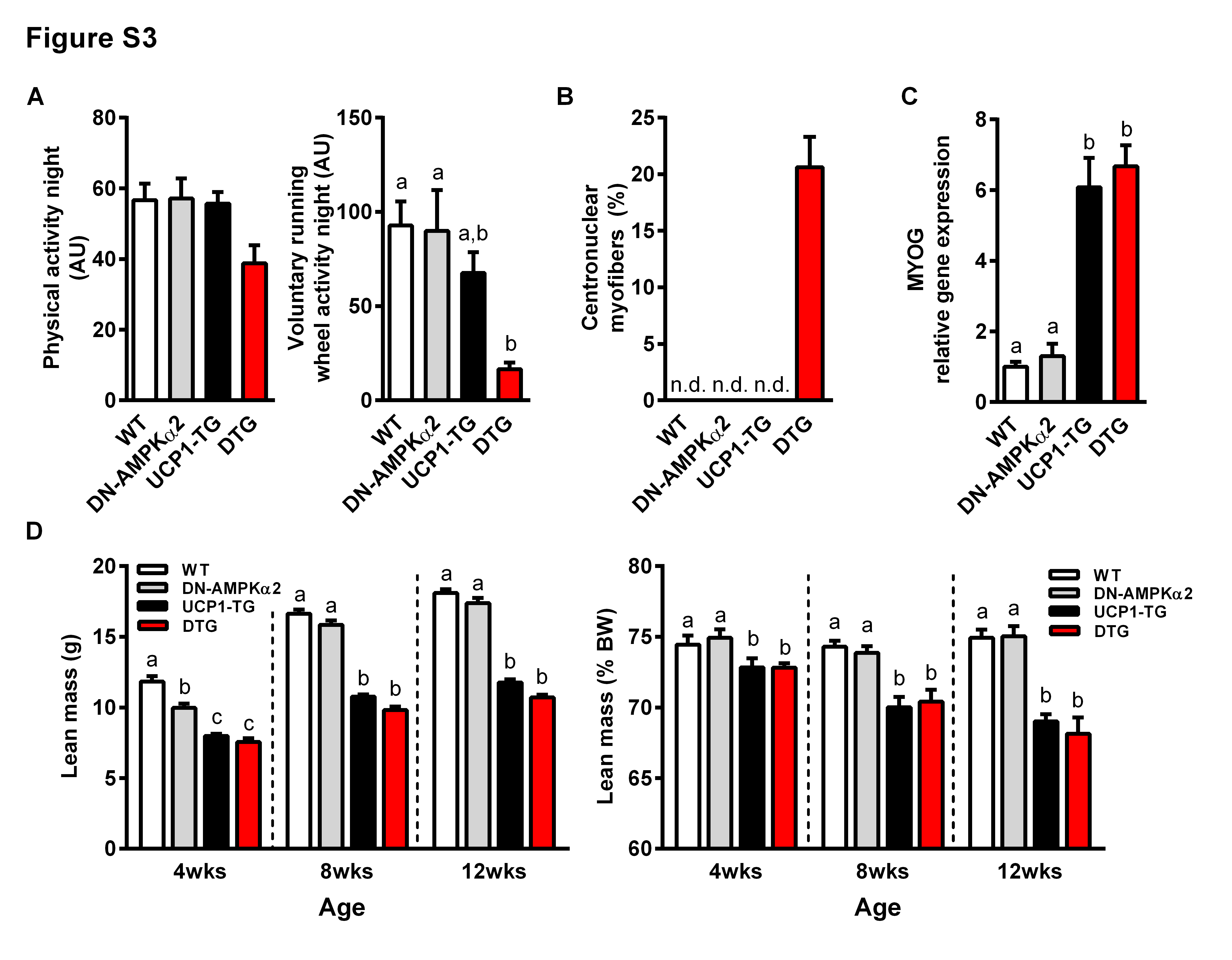

Supplement: Figure S3 — Characterization of in muscle function, myofiber morphology, myogenesis and early lean mass development. (A) Quantification of locomotor activity during night at 11 wks of age (n = 6–10 per group). (B) Percentage of centronuclear myofibers of M. tibialis anterior (TA) muscle from a 20-wk-old WT, a DN-AMPKα2, a UCP1-TG and a DTG mouse (n = 3 per group). (C) Gene expression analysis of myogenesis marker MYOG in Quadriceps muscle of 12-wk-old WT, DN-AMPKα2, UCP1-TG and DTG mice by quantitative RT-PCR (n = 8 per group). (D) Means with different letters are significantly different (1way ANOVA and Bonferroni's multiple comparisons test, p<0.05). (TIF) [file pone.0094689.s003.tif]

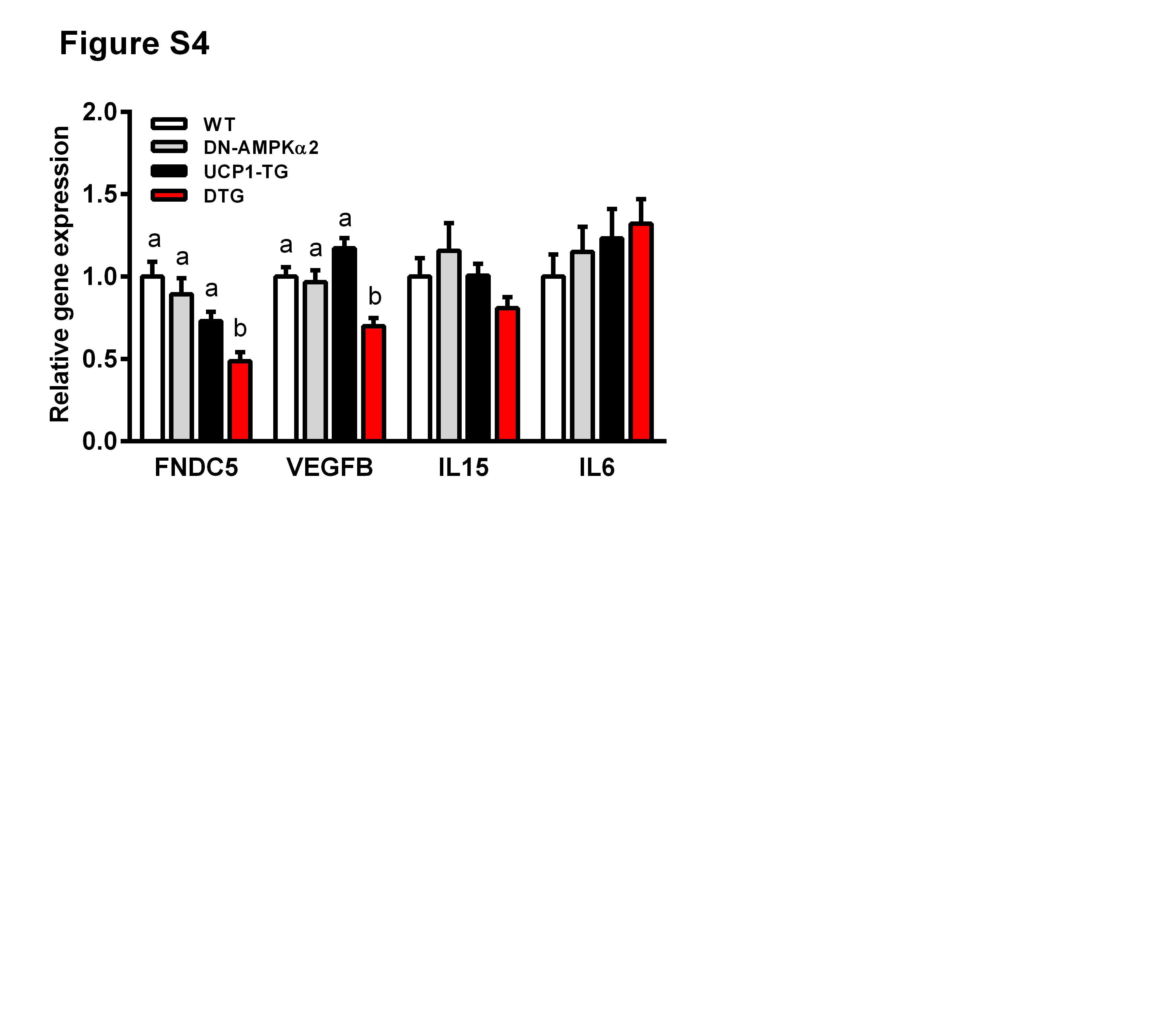

Supplement: Figure S4 — Decreased FNDC5 (irisin) and VEGFB myokine expression in SM of DTG mice. Gene expression analysis of selected myokines in Quadriceps muscle of 12-wk-old WT, DN-AMPKα2, UCP1-TG and DTG mice by quantitative RT-PCR (n = 8 per group). Means with different letters are significantly different (1way ANOVA and Bonferroni's multiple comparisons test, p<0.05). (TIF) [file pone.0094689.s004.tif]

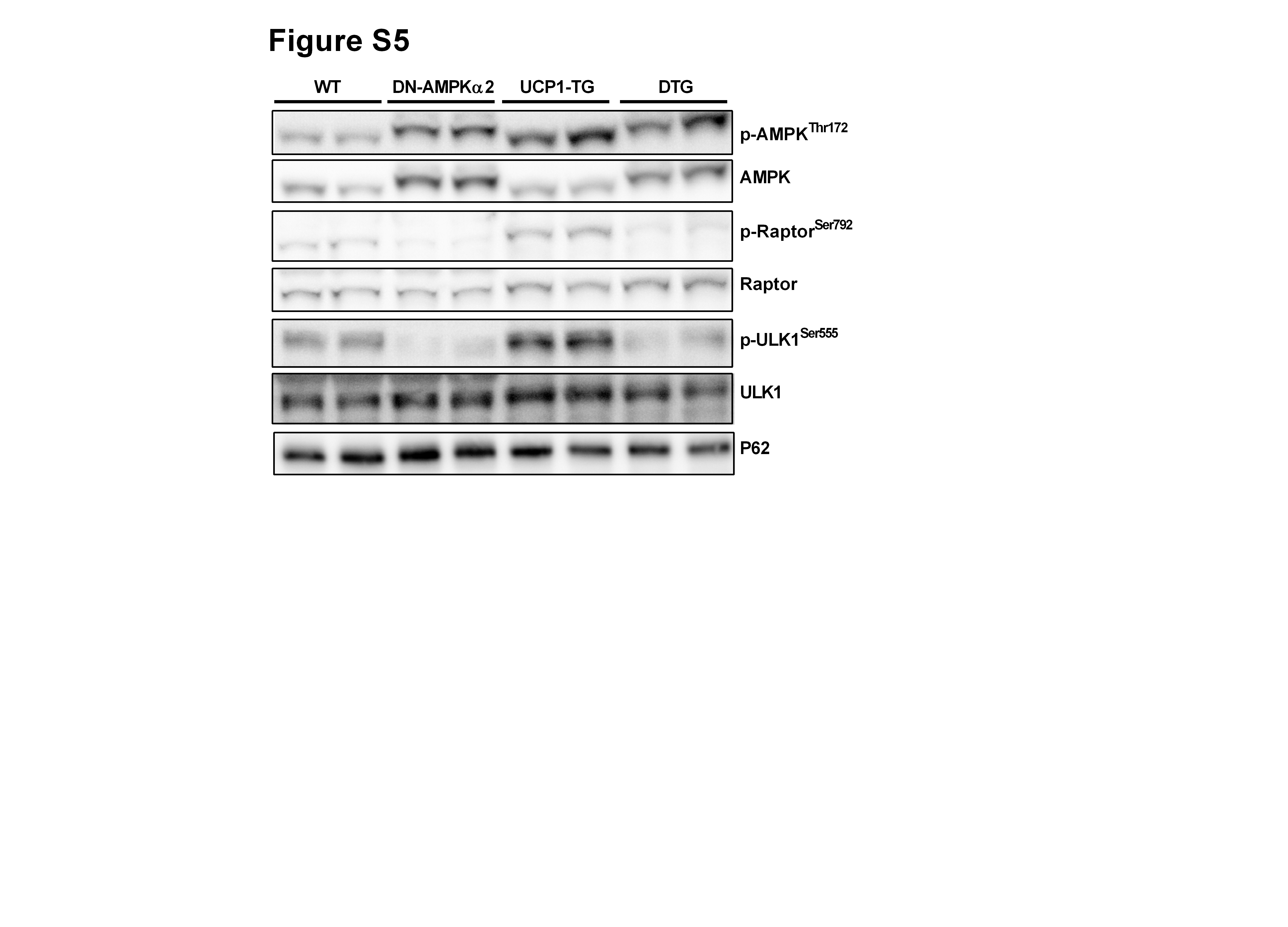

Supplement: Figure S5 — Suppressed induction of AMPK downstream targets important for mitophagy regulation in SM of DTG mice. Representative western blot showing expression of key proteins (and AMPK targets) involved in mitophagy regulation in Quadriceps muscle from 12-wk-old WT, DN-AMPKα2, UCP1-TG and DTG mice, Mitofusin-2 (MFN2) was used as a loading control (n = 2 out of 6-8 analyzed per group). (TIF) [file pone.0094689.s005.tif]

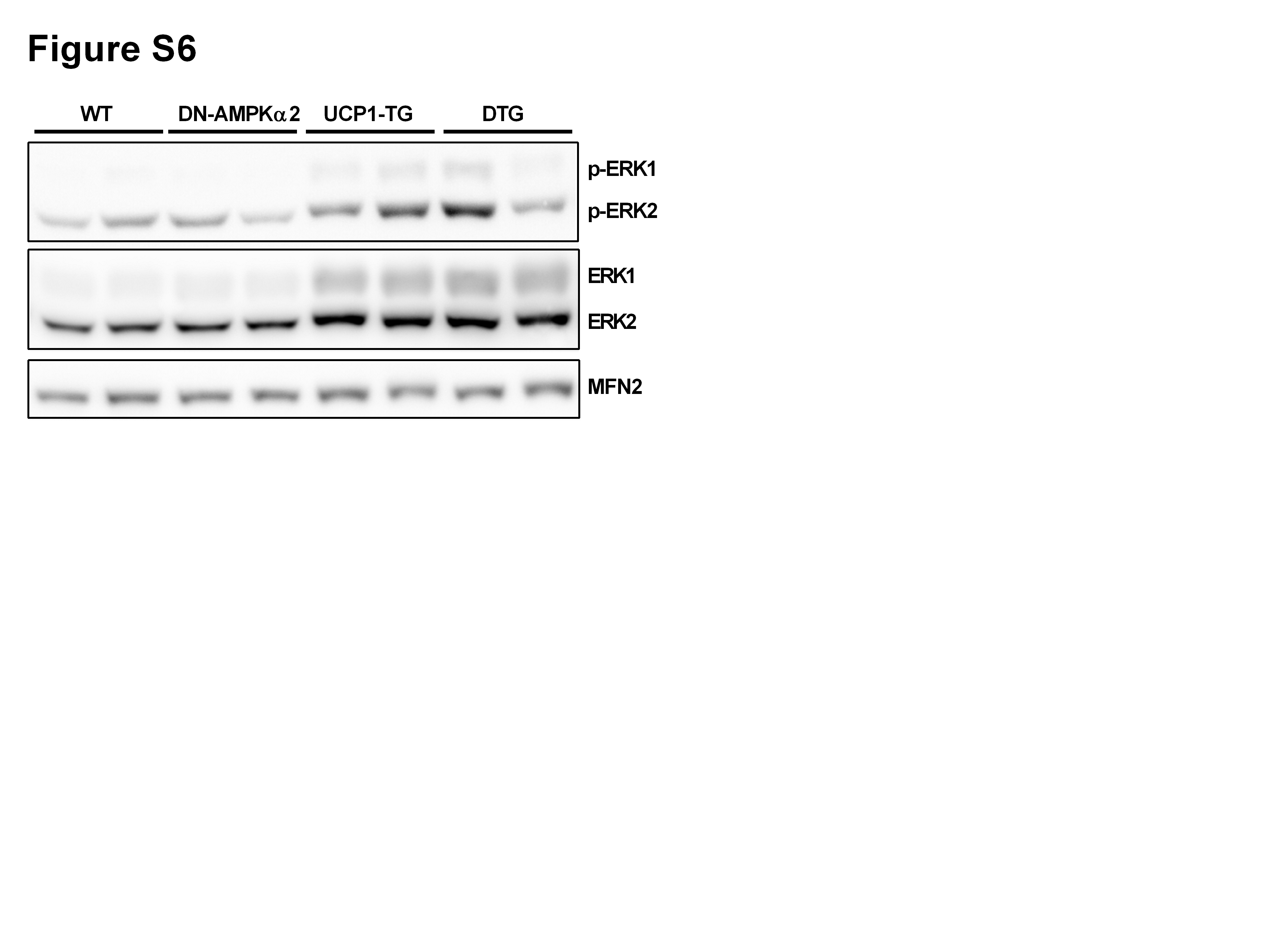

Supplement: Figure S6 — Activation ERK2 signaling in SM of UCP1-TG and DTG mice. Representative western blot of Quadriceps muscle from 12-wk-old WT, DN-AMPKα2, UCP1-TG and DTG mice, Mitofusin-2 (MFN2) was used as a loading control (n = 2 out of 6-8 analyzed per group). (TIF) [file pone.0094689.s006.tif]
